# Supplementary material for: Reduction of Cystatin B results in increased cathepsin B activity in disomic but not Trisomy 21 human cellular and mouse models
Source: PLoS One. 2025 Jan 22;20(1):e0316822. doi: 10.1371/journal.pone.0316822 (PMC11753708; doi:10.1371/journal.pone.0316822)
Supplement: S1 Fig — (PDF) [file pone.0316822.s001.pdf]

For Figure 1

PVDF membranes

By Odyssey CLx imaging system (Yixing Wu)

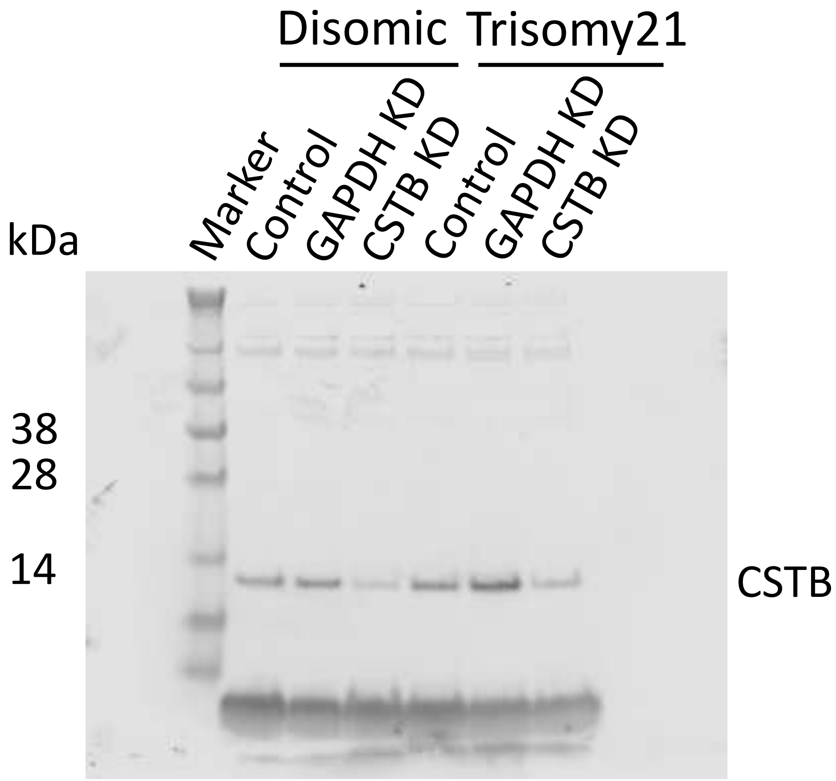

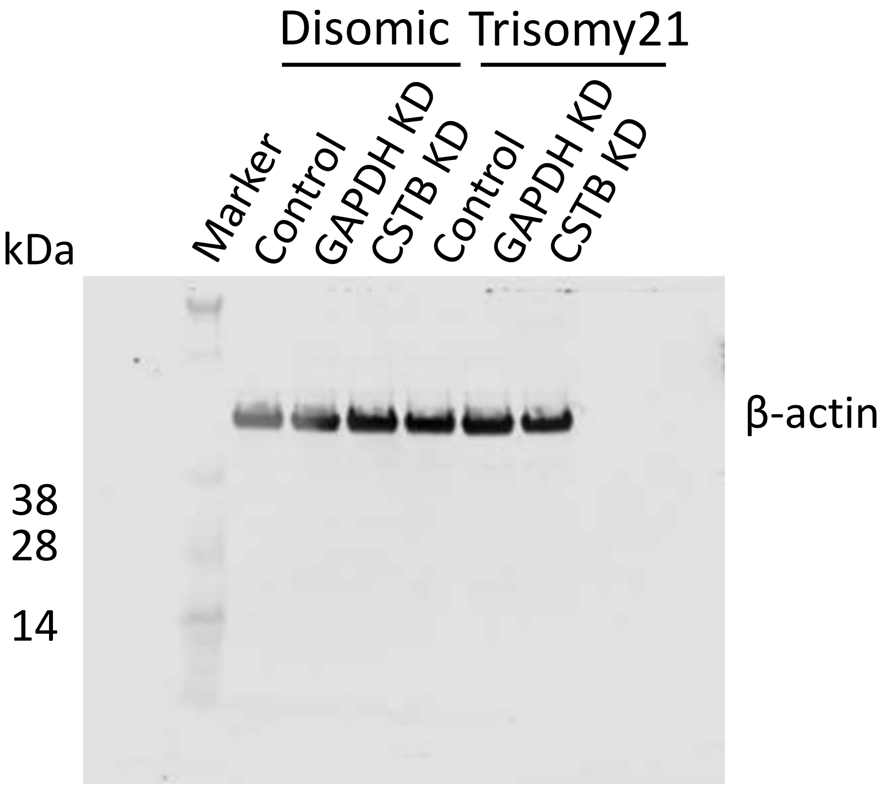

For Figure 1

PVDF membranes

By Odyssey CLx imaging system (Yixing Wu)

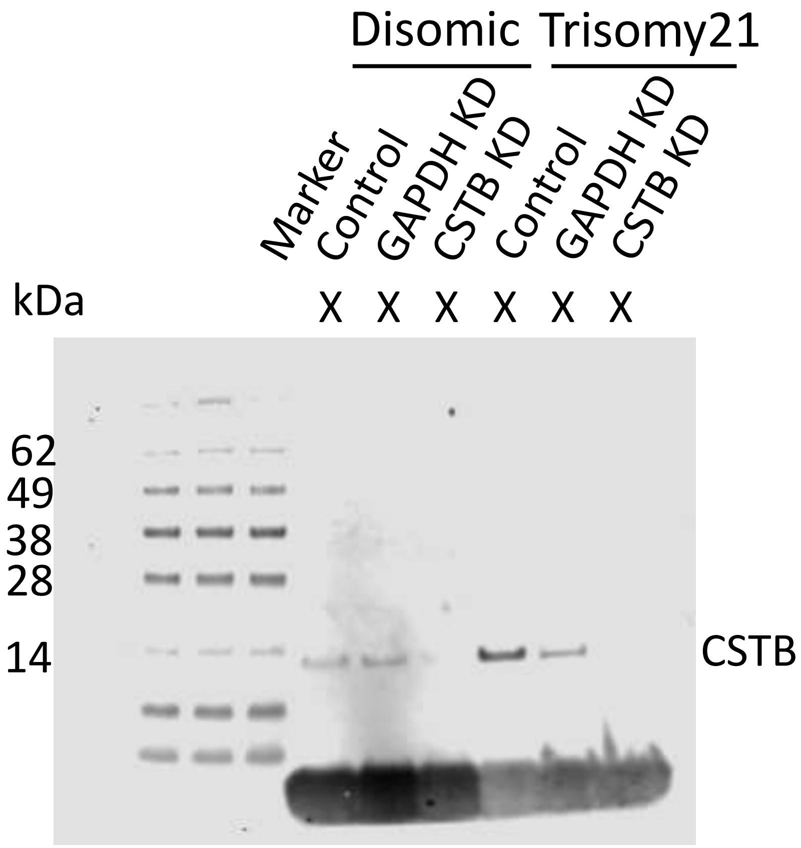

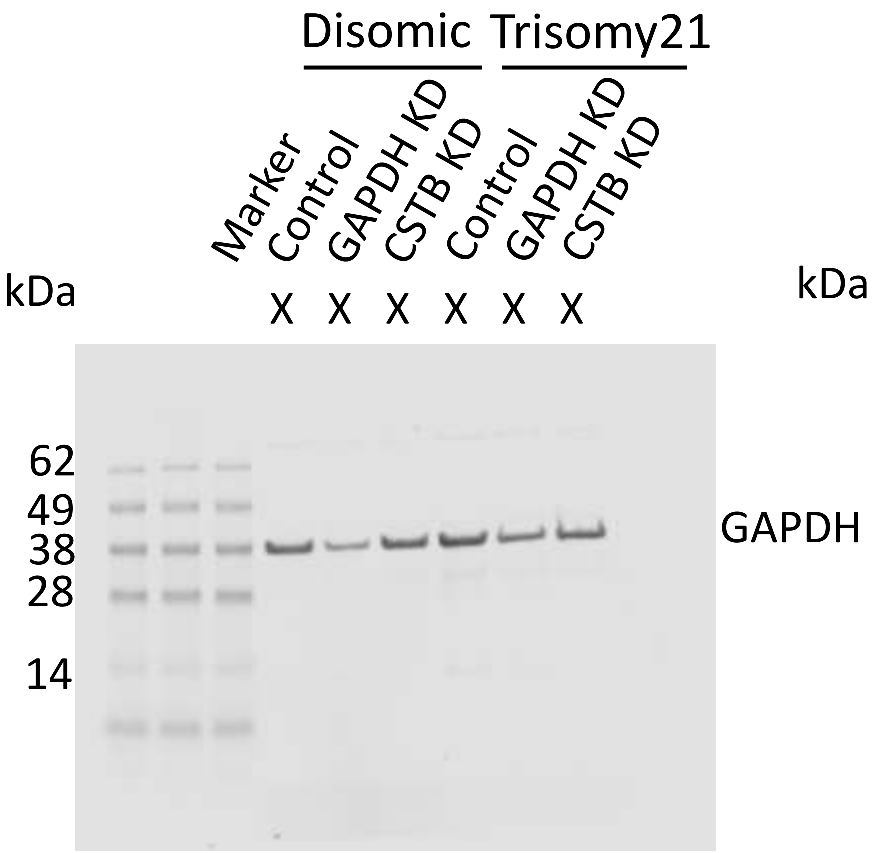

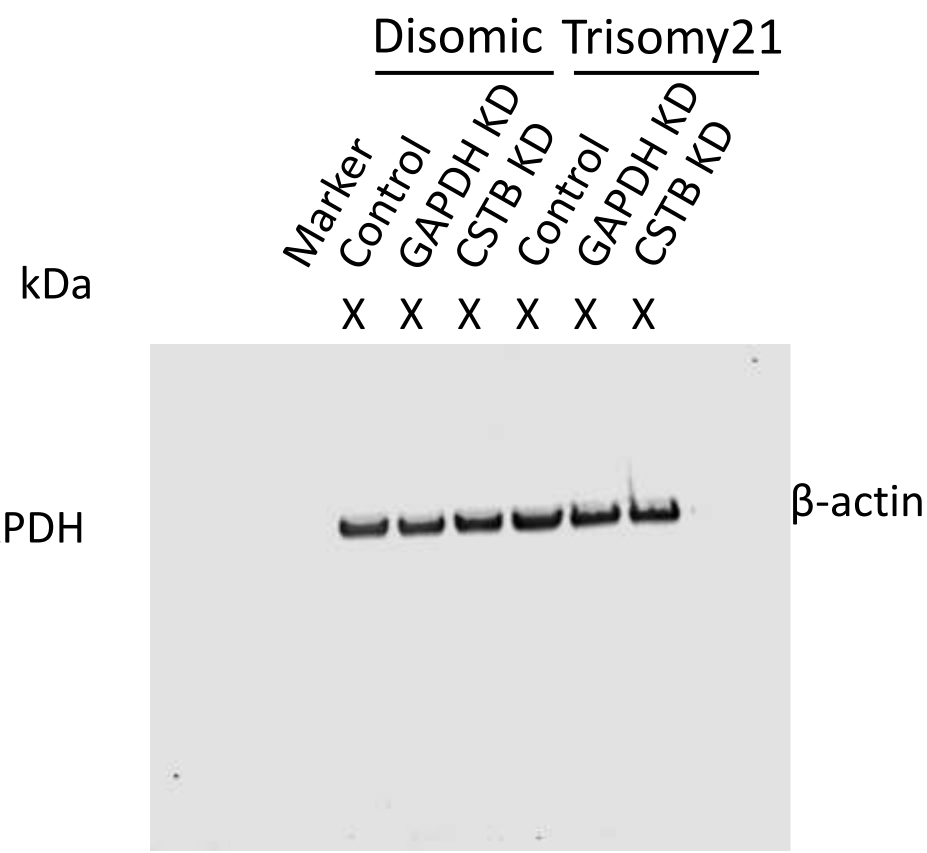

For Figure 1

PVDF membranes

By Odyssey CLx imaging system (Yixing Wu)

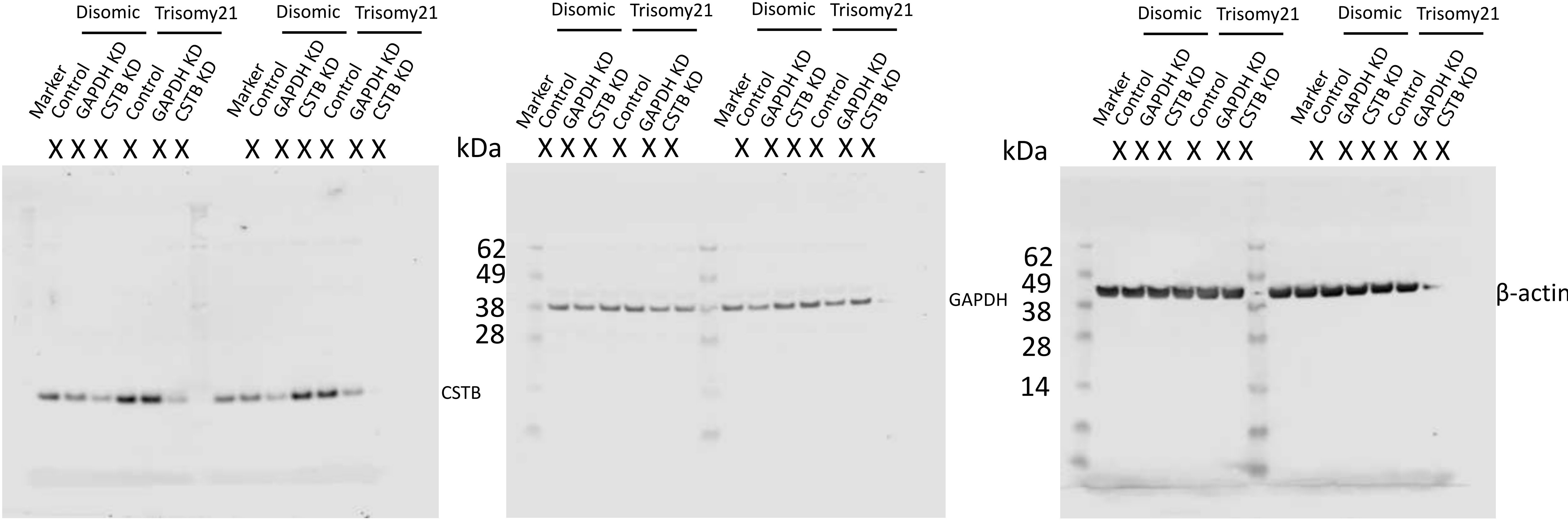

X – indicates lanes from which data was quantitated but that were not included in the representative images in Wu et al 2024.

For Figure 1

PVDF membranes

PVDF membranes

By Odyssey CLx imaging system (Yixing Wu)

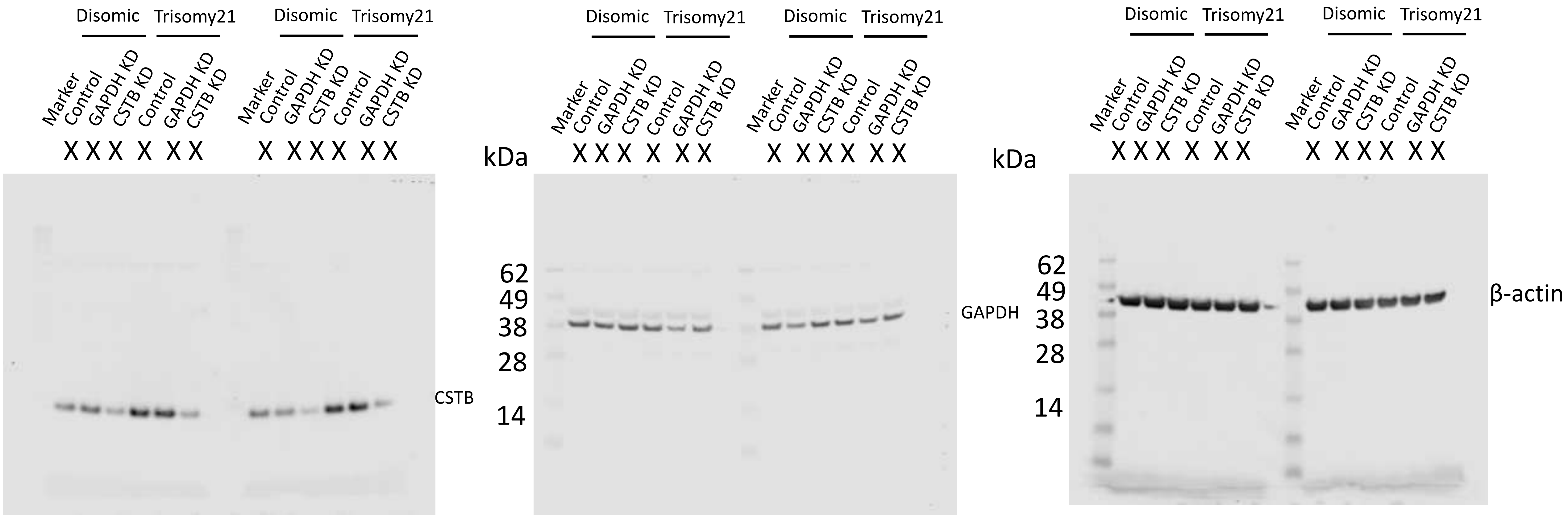

X – indicates lanes from which data was quantitated but that were not included in the representative images in Wu et al 2024.

For Figure 1

PVDF membranes

By Odyssey CLx imaging system (Yixing Wu)

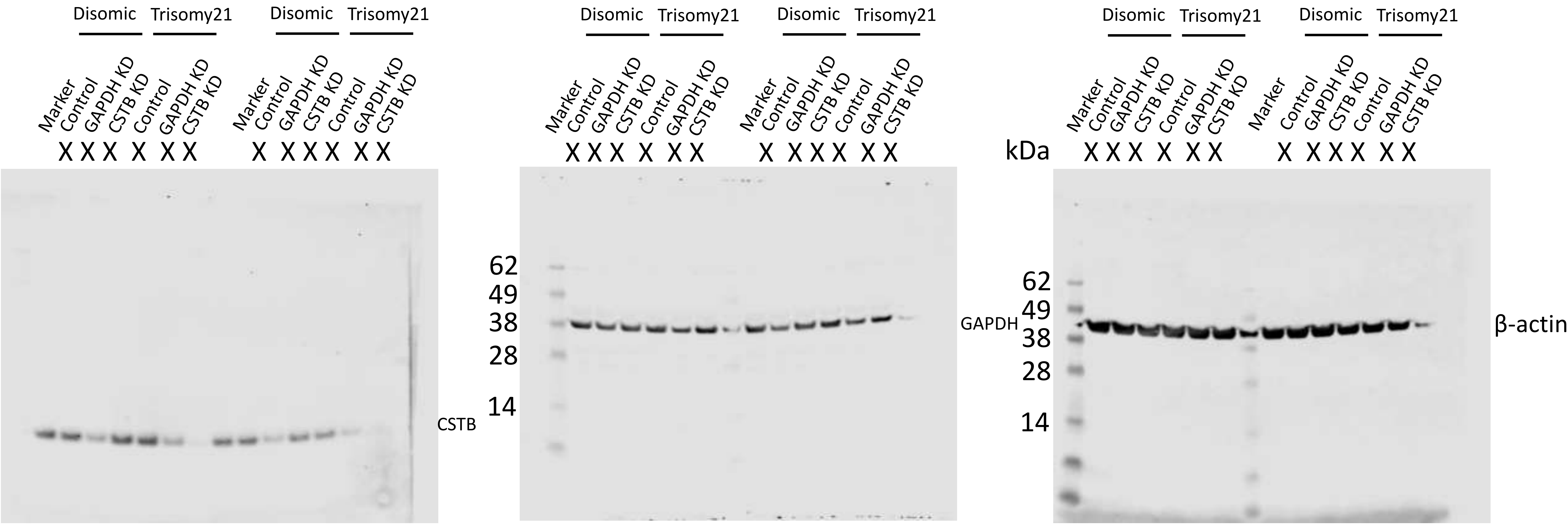

X – indicates lanes from which data was quantitated but that were not included in the representative images in Wu et al 2024.

For Figure 1

PVDF membranes

By Odyssey CLx imaging system (Yixing Wu)

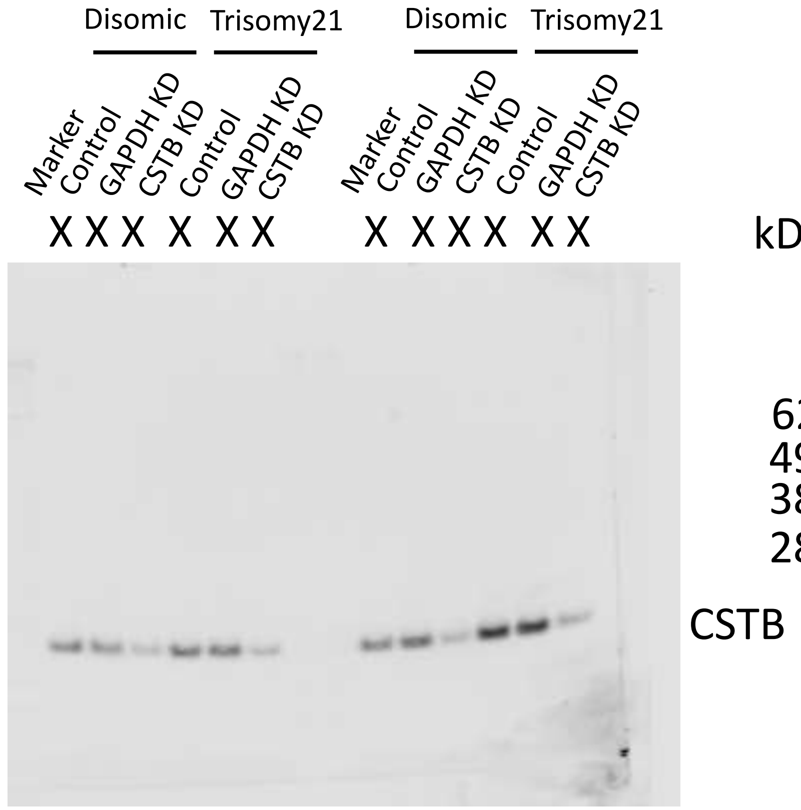

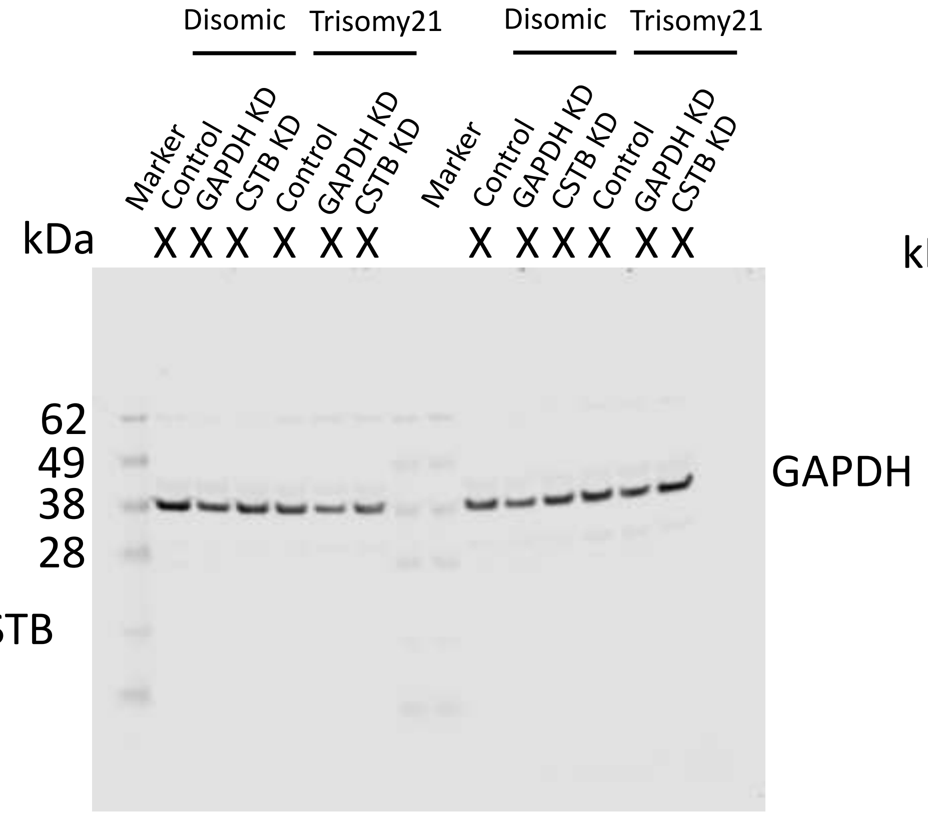

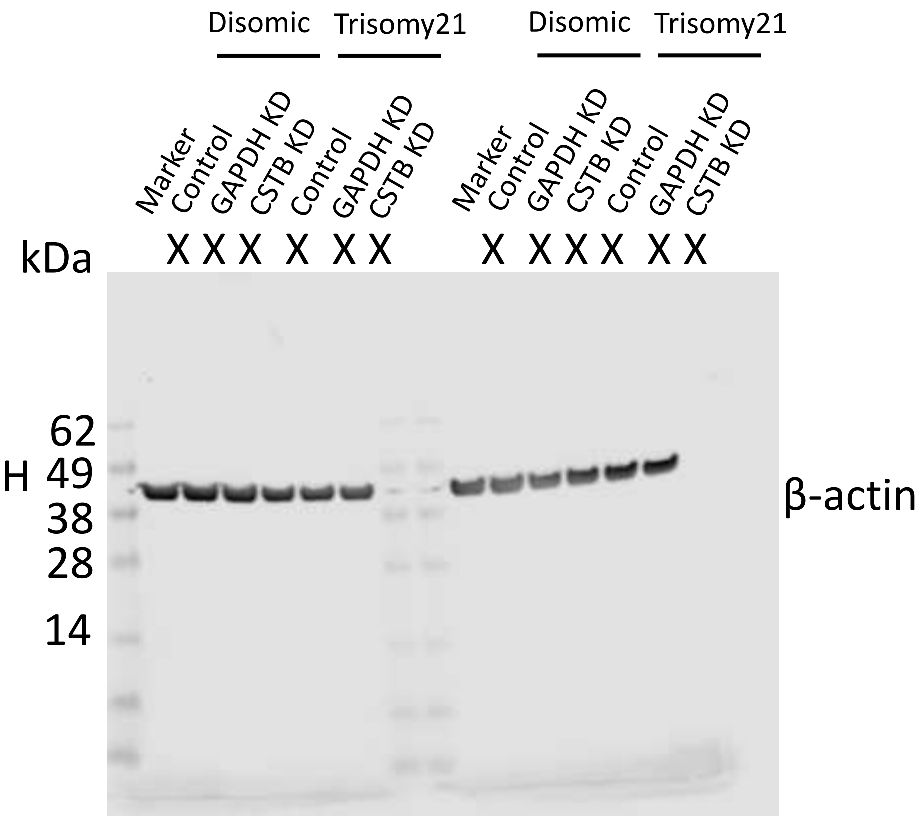

X – indicates lanes from which data was quantitated but that were not included in the representative images in Wu et al 2024.

For Figure 1

PVDF membranes

By ChemiDoc Imaging System (Yixing Wu)

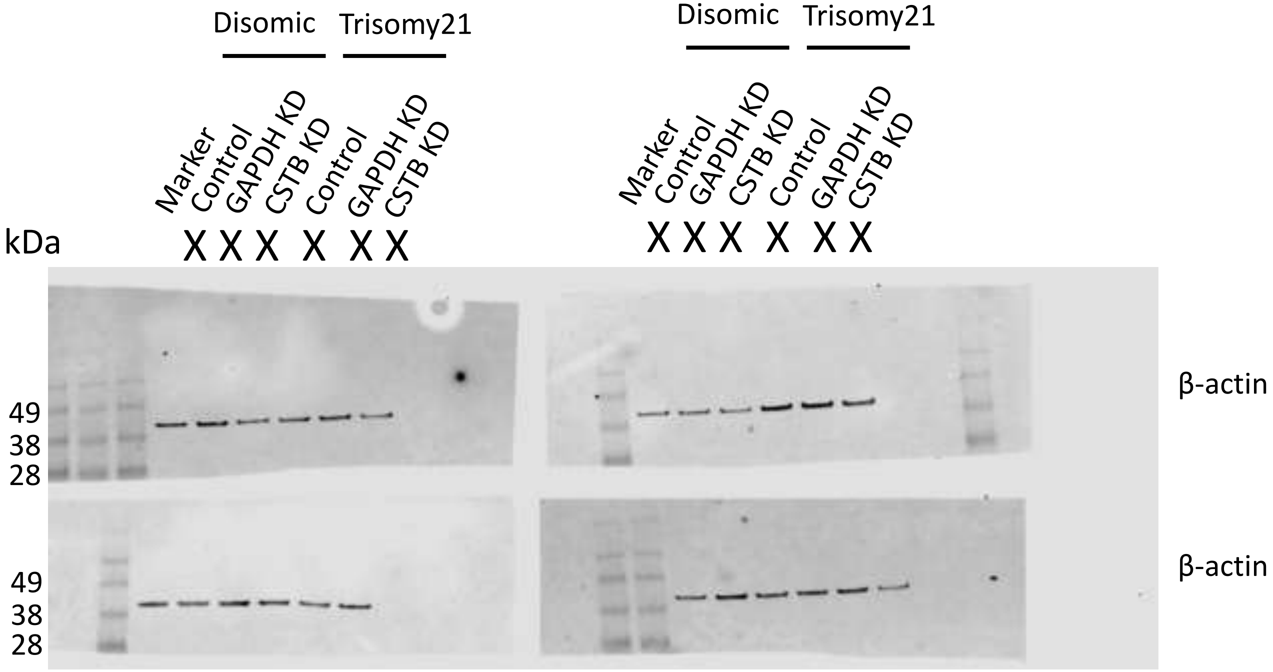

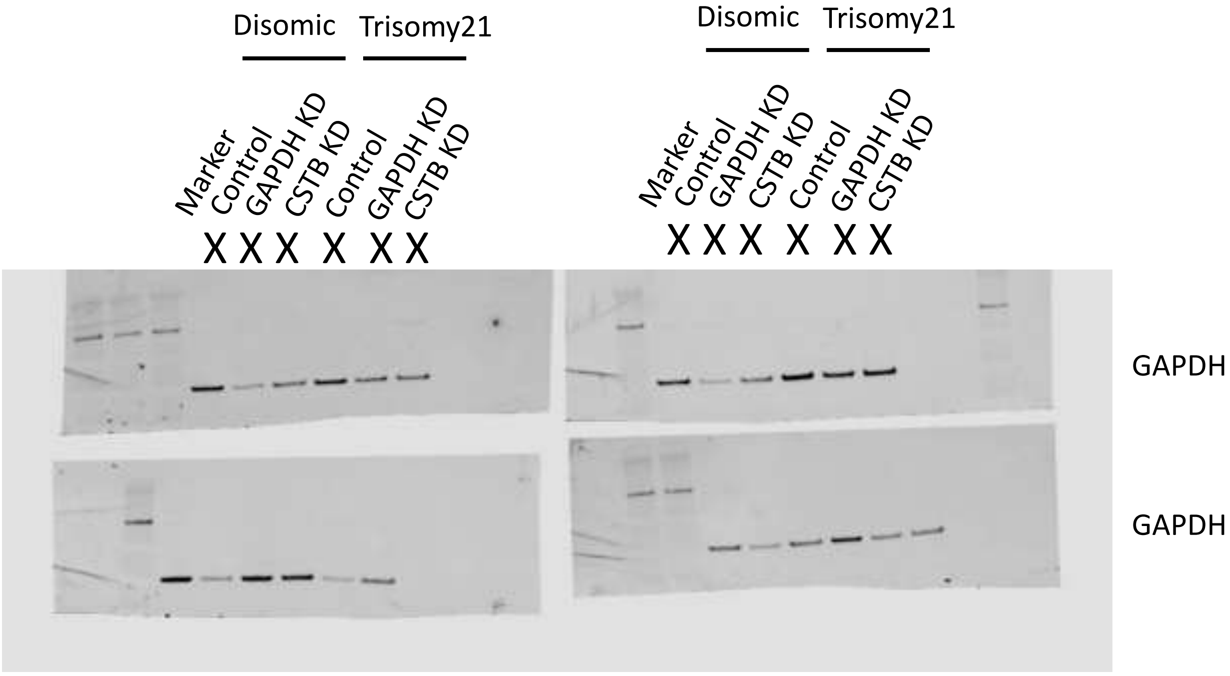

X – indicates lanes from which data was quantitated but that were not included in the representative images in Wu et al 2024.

For Figures 1a and 2a

PVDF membranes

By Odyssey CLx imaging system (Yixing Wu)

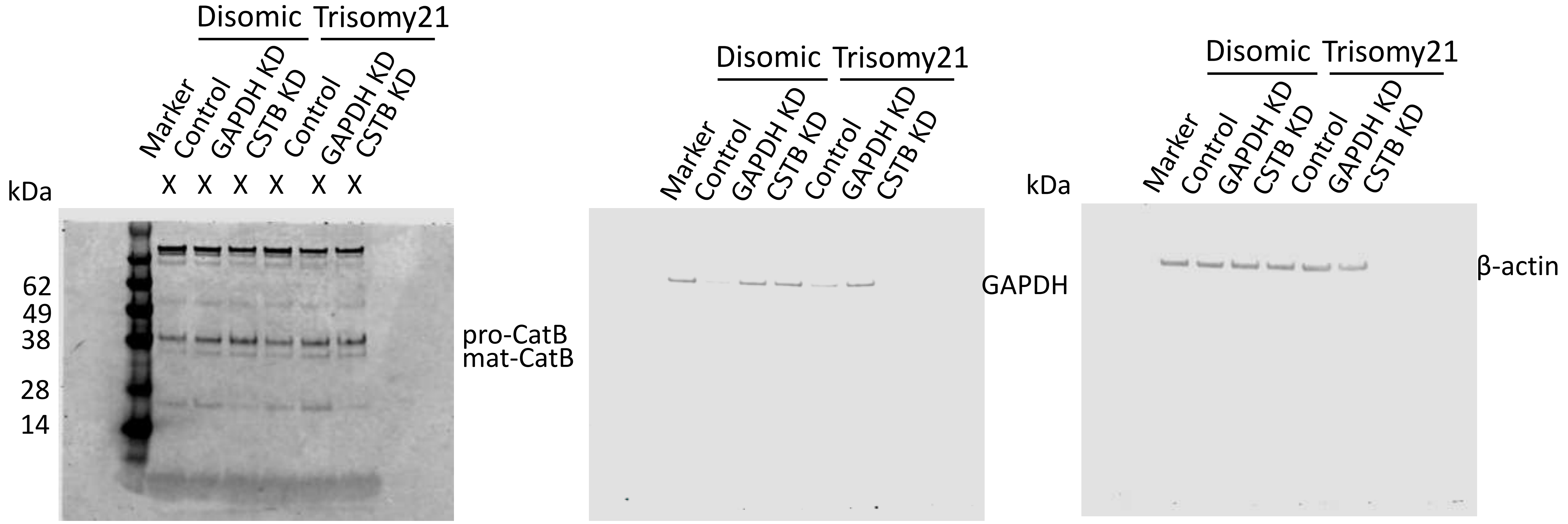

X – indicates lanes from which data was quantitated but that were not included in the representative images in Wu et al 2024.

For Figures 1a and 2a

PVDF membranes

By Odyssey CLx imaging system (Yixing Wu)

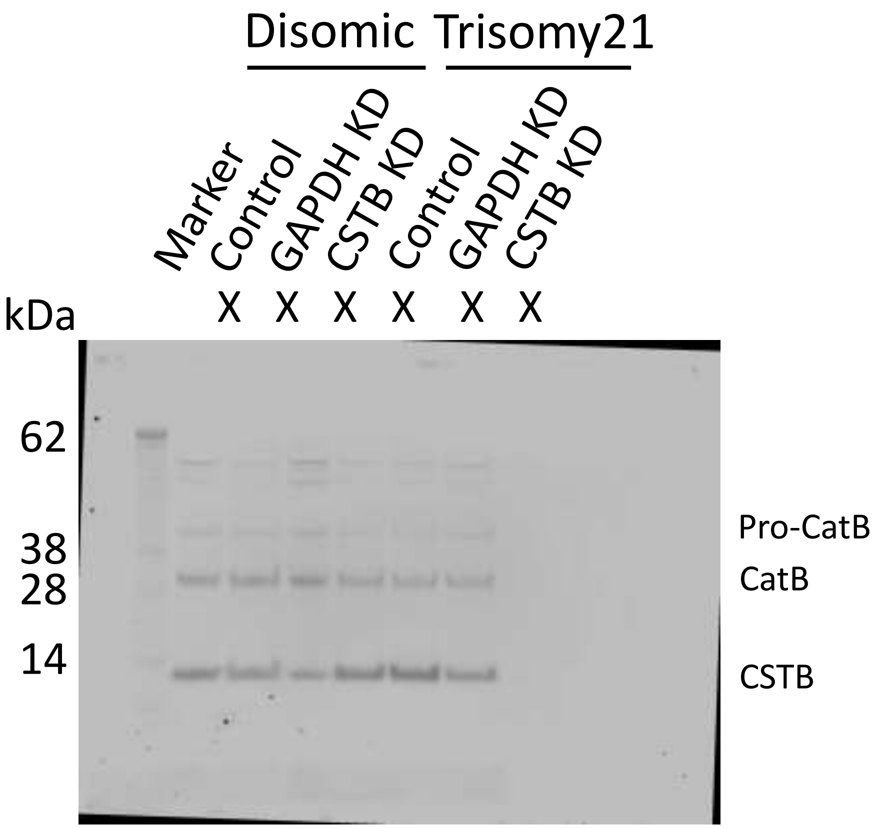

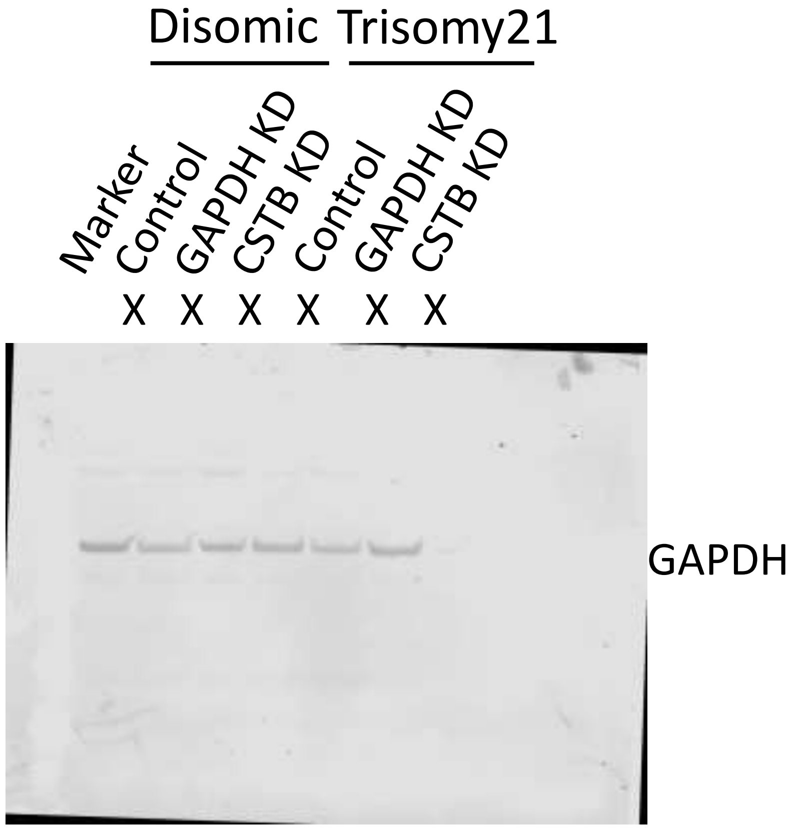

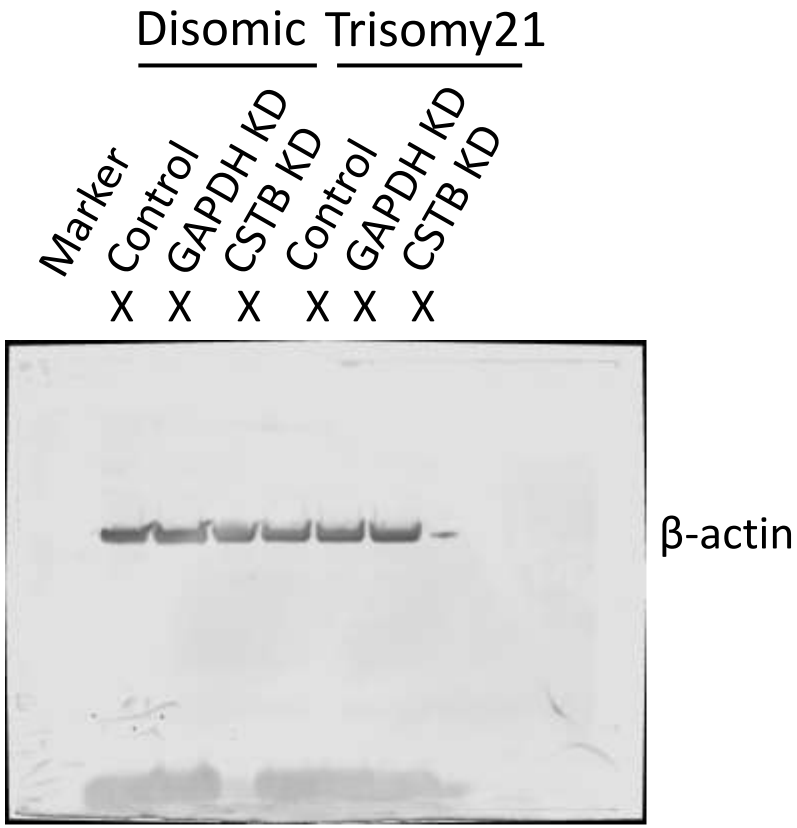

X – indicates lanes from which data was quantitated but that were not included in the representative images in Wu et al 2024.

For Figures 1a and 2a

PVDF membranes

By Odyssey CLx imaging system (Yixing Wu)

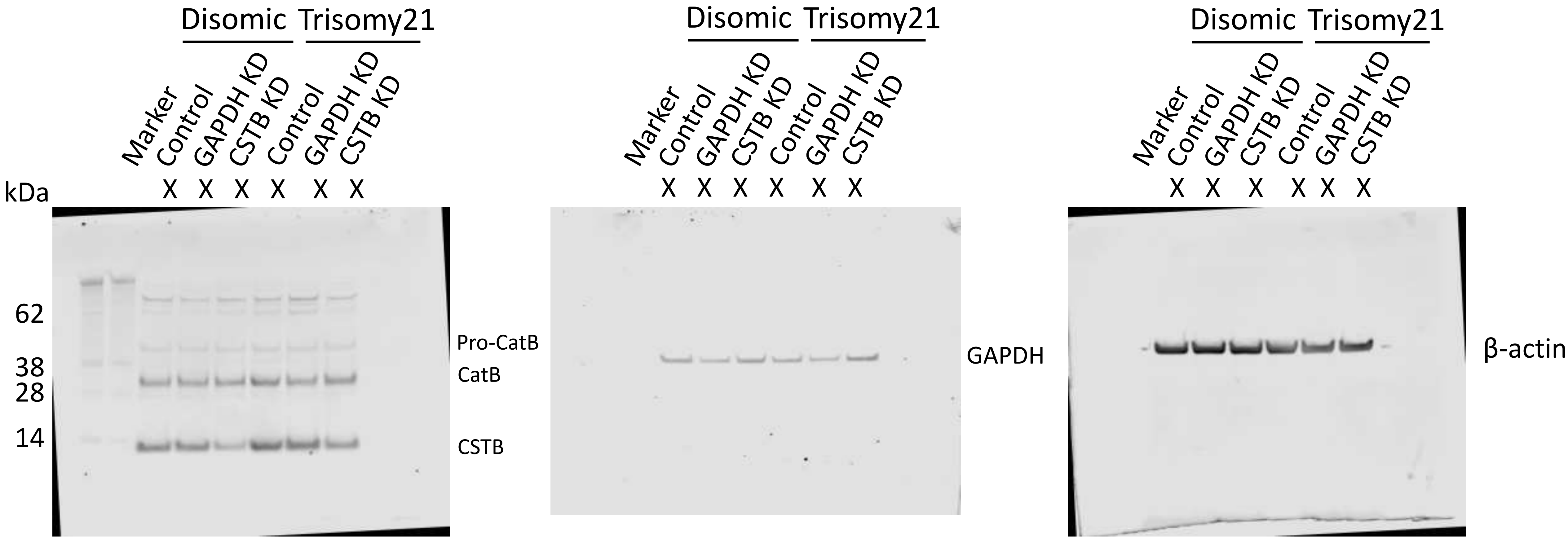

X – indicates lanes from which data was quantitated but that were not included in the representative images in Wu et al 2024.

For Figure 2a

PVDF membranes

By Odyssey CLx imaging system (Yixing Wu)

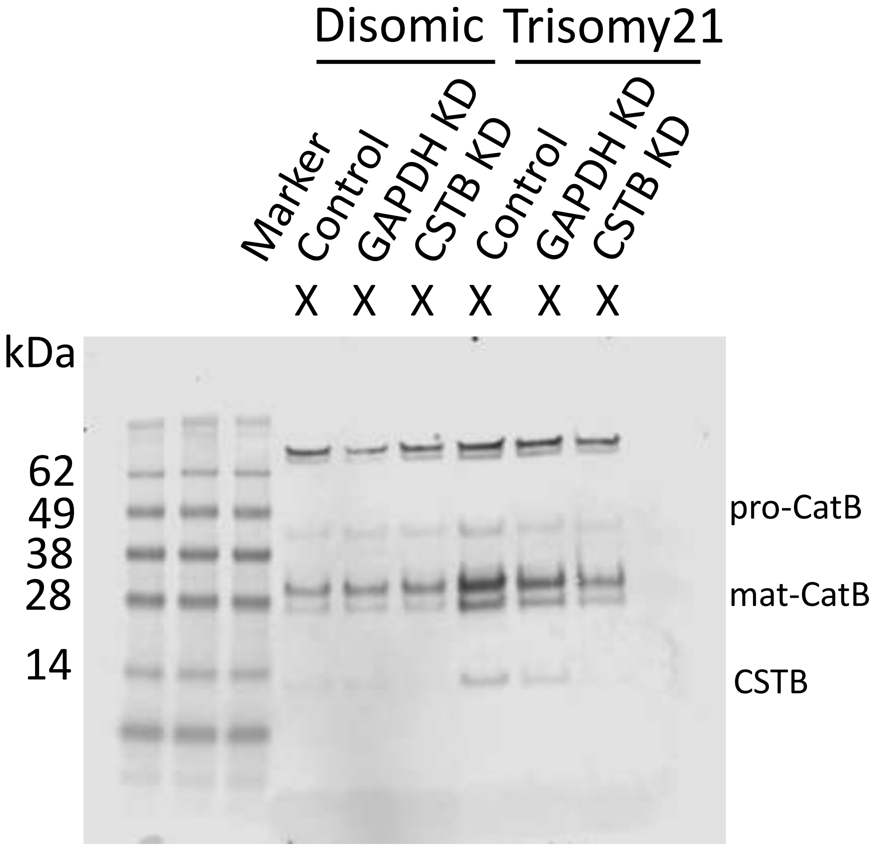

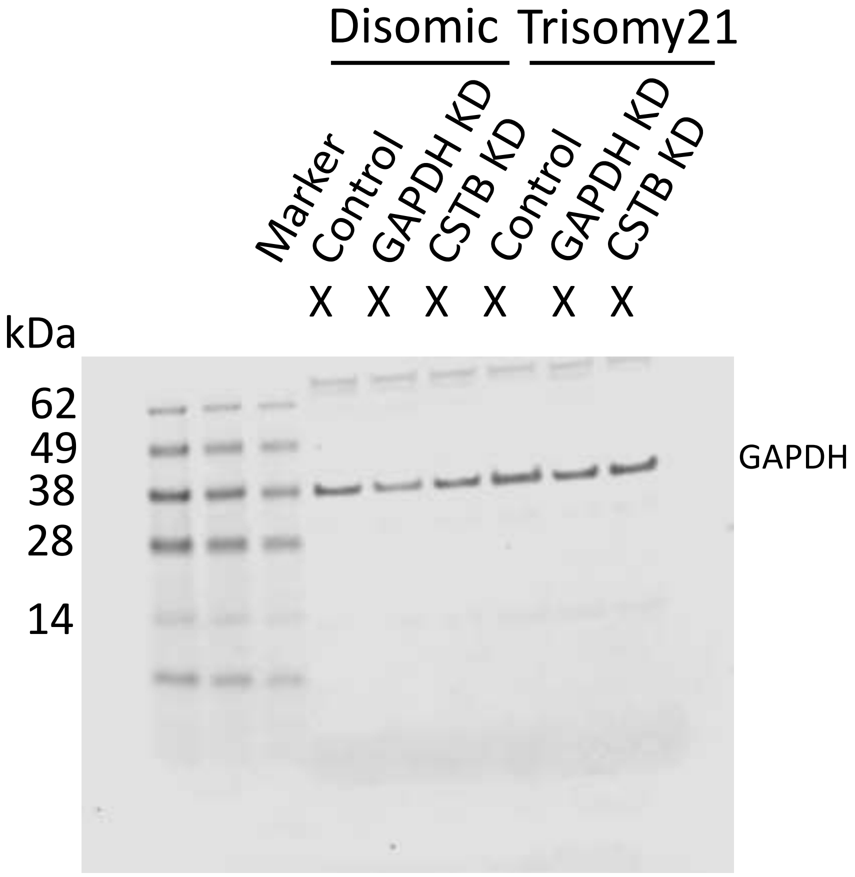

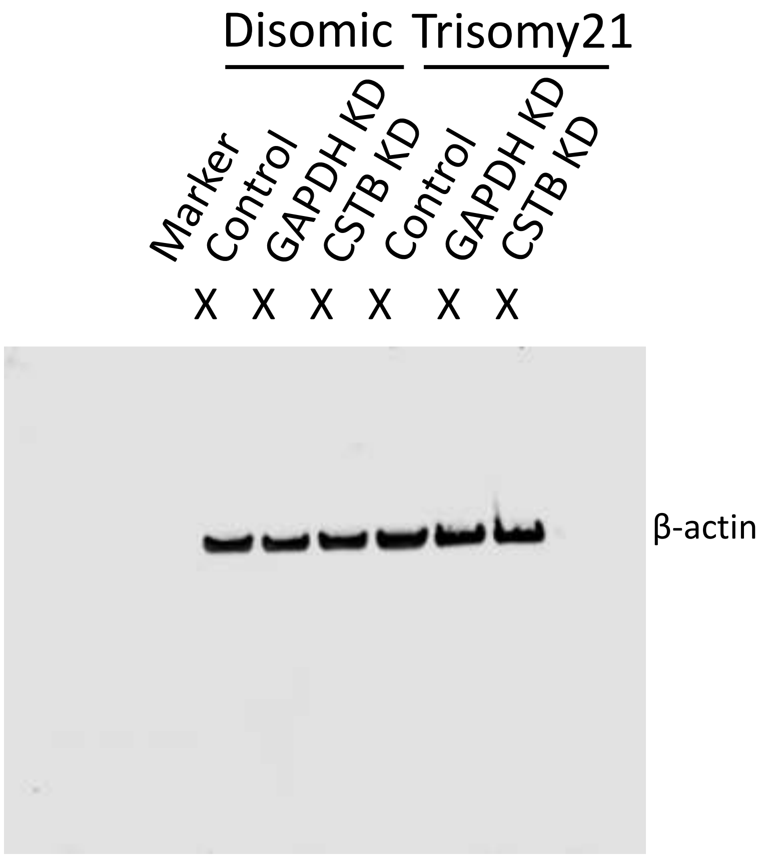

The same blot as the β-actin blot for CSTB n1 (Figure 1)

X – indicates lanes from which data was quantitated but that were not included in the representative images in Wu et al 2024.

For Figures 1a and 2a

PVDF membranes

By Odyssey CLx imaging system (Yixing Wu)

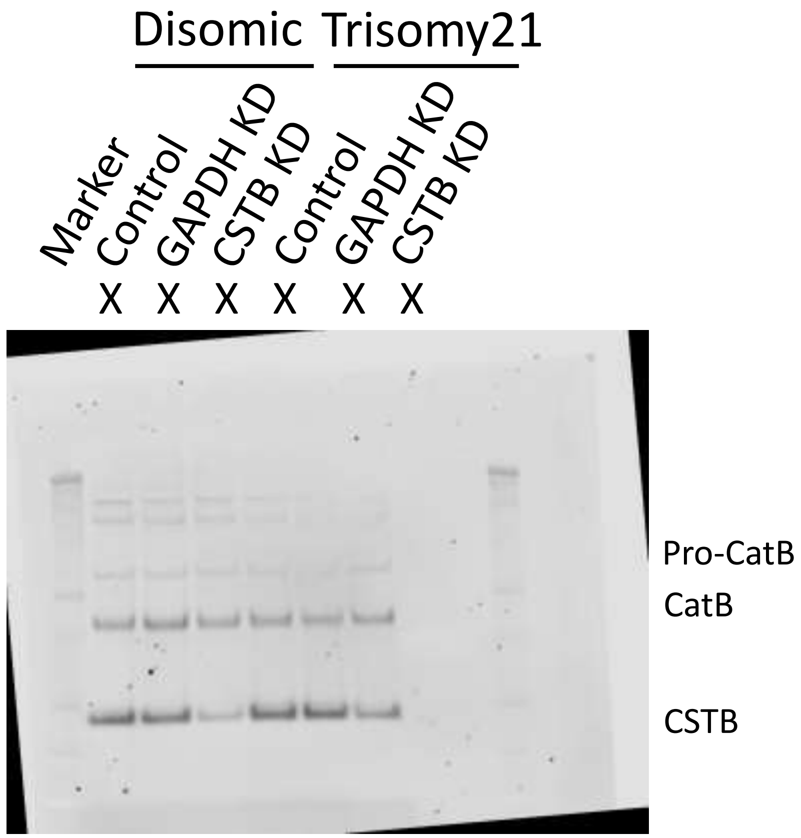

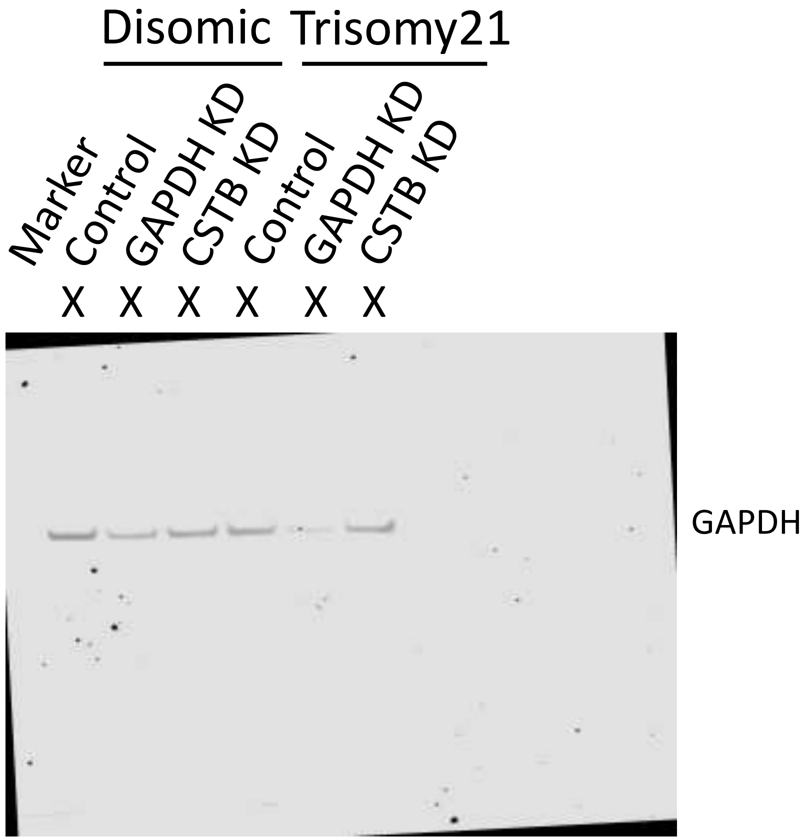

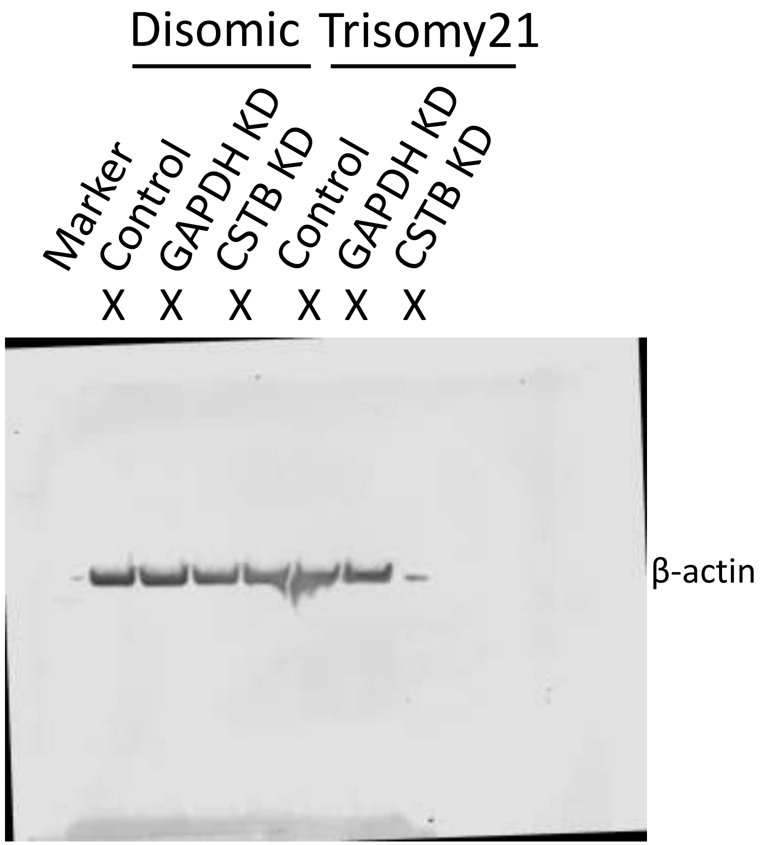

X – indicates lanes from which data was quantitated but that were not included in the representative images in Wu et al 2024.

For Figures 1a and 2a

PVDF membranes

By Odyssey CLx imaging system (Yixing Wu)

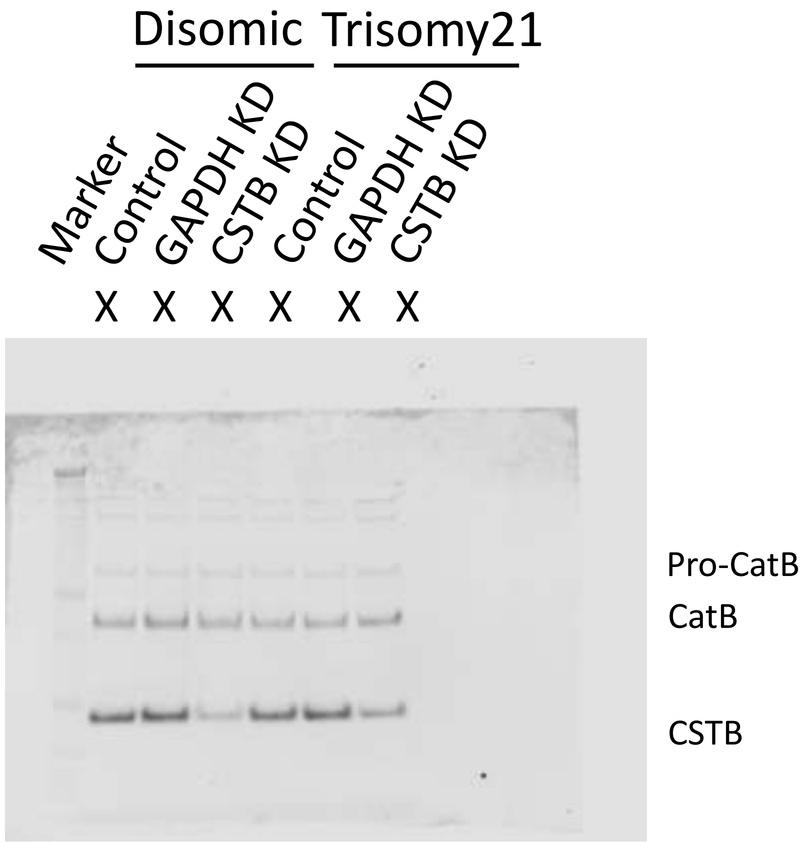

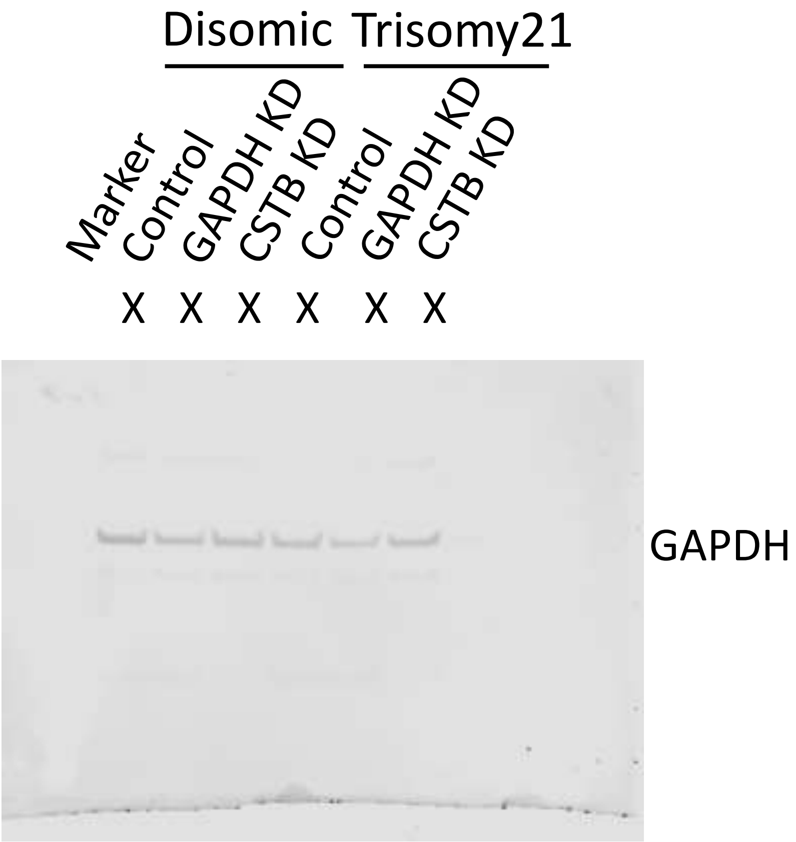

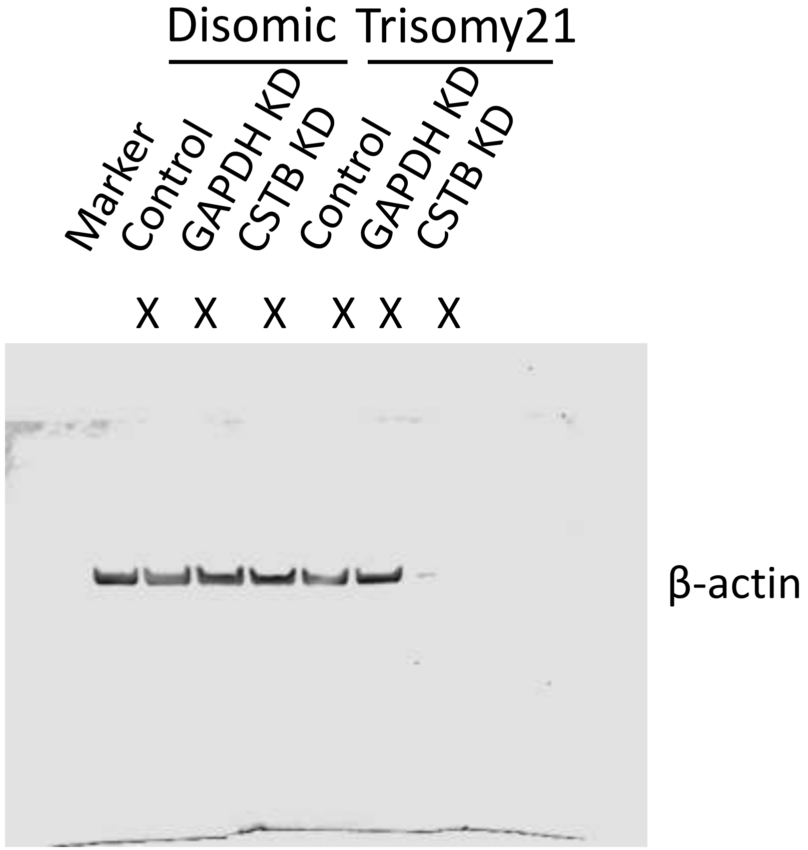

X – indicates lanes from which data was quantitated but that were not included in the representative images in Wu et al 2024.

For Figure 2a

PVDF membranes

By Odyssey CLx imaging system (Yixing Wu)

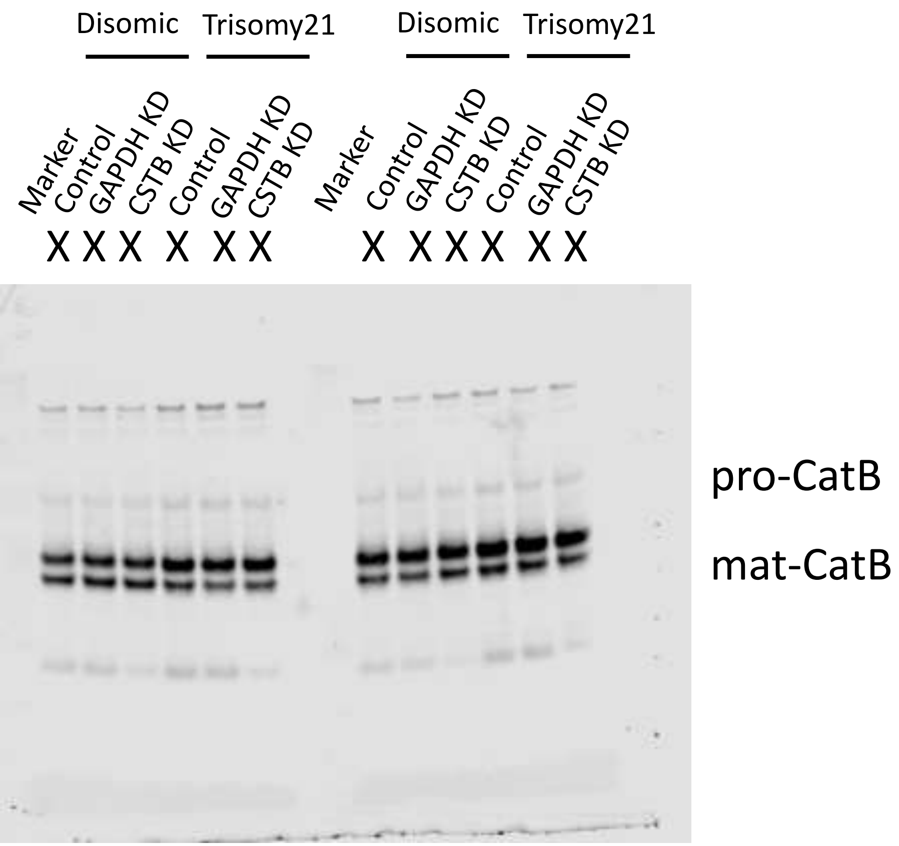

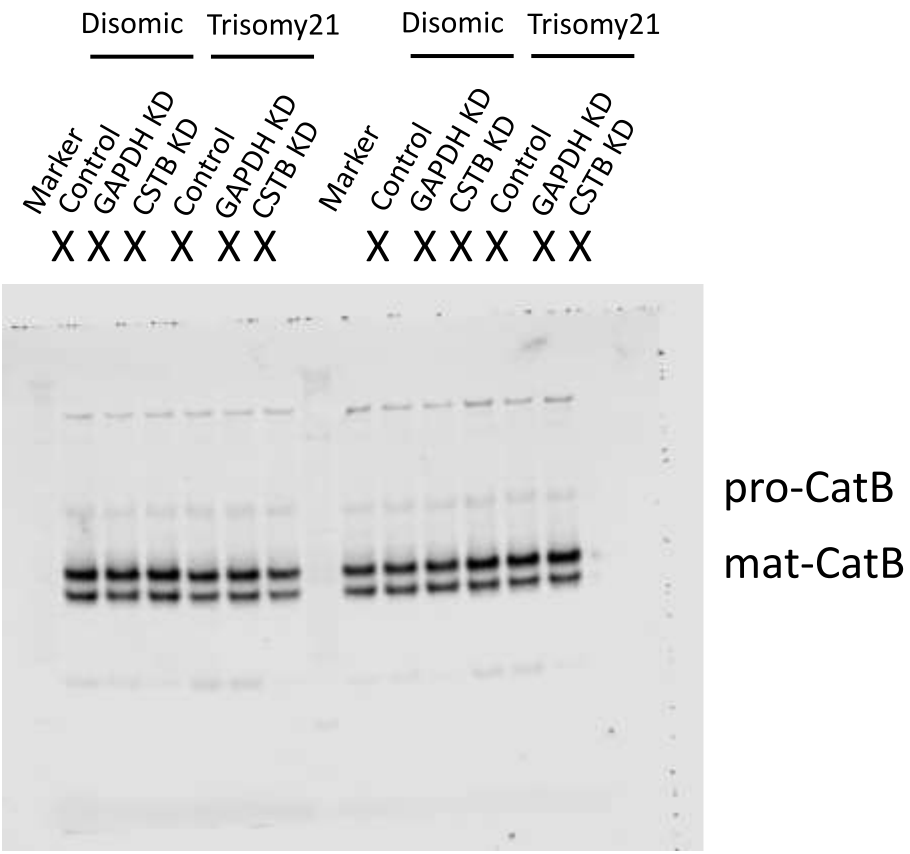

For Figure 2a

PVDF membranes

By Odyssey CLx imaging system (Yixing Wu)

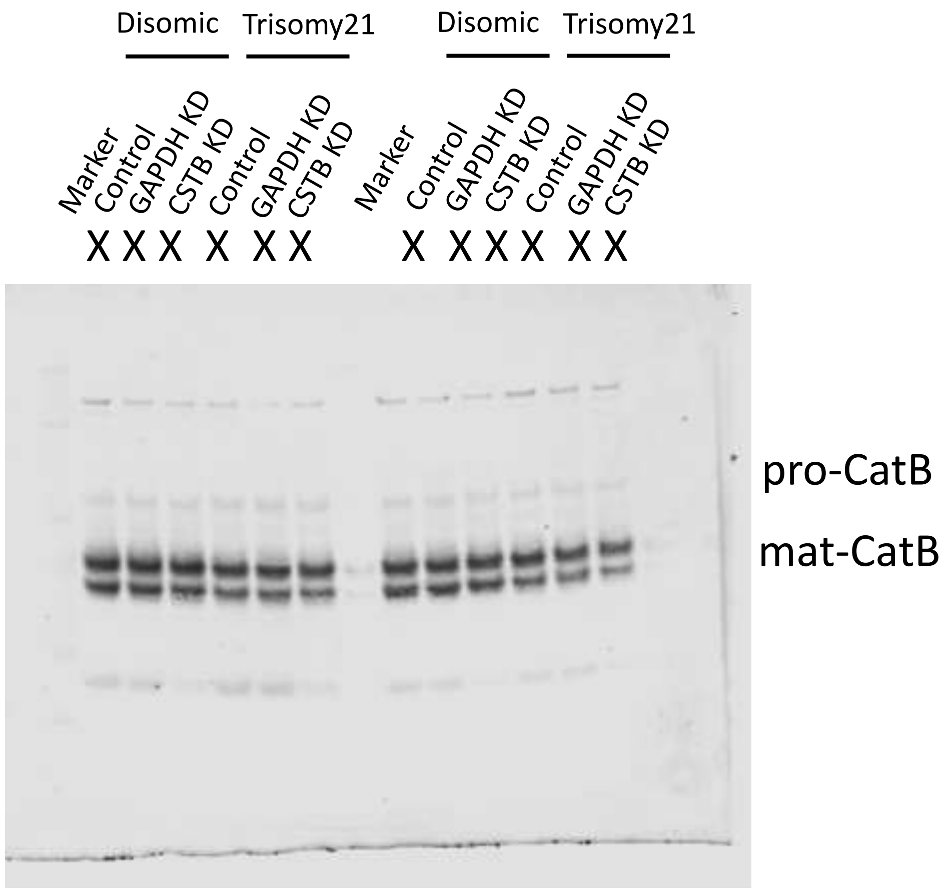

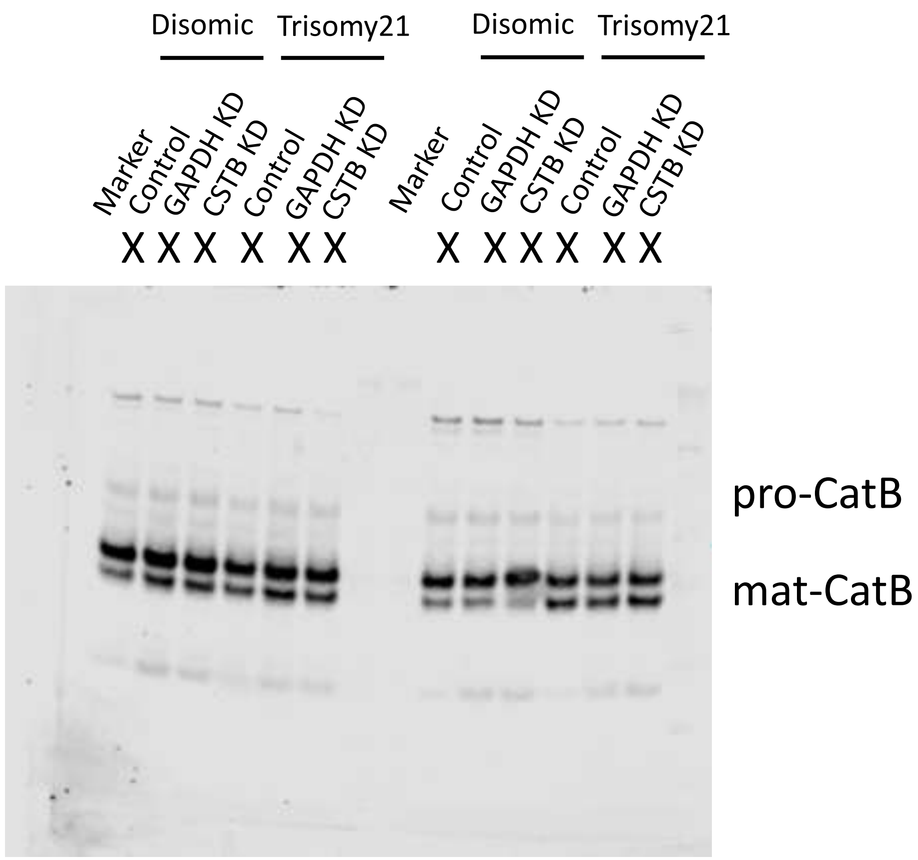

For Figure 3a

PVDF membranes

By Odyssey CLx imaging system (Yixing Wu)

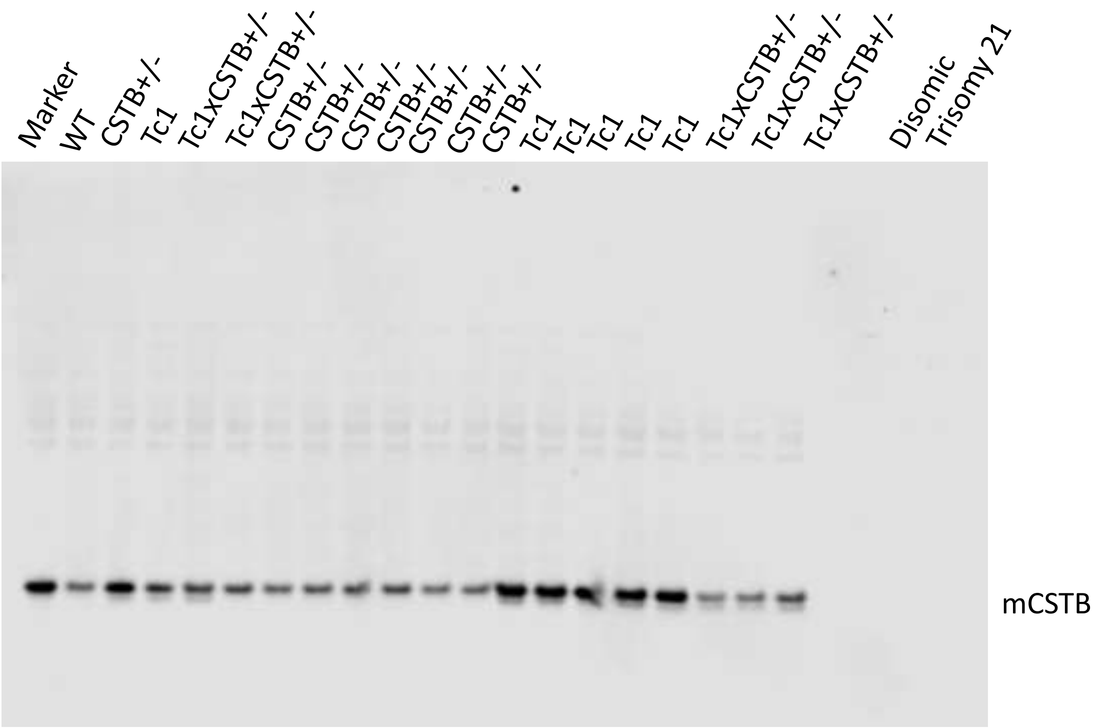

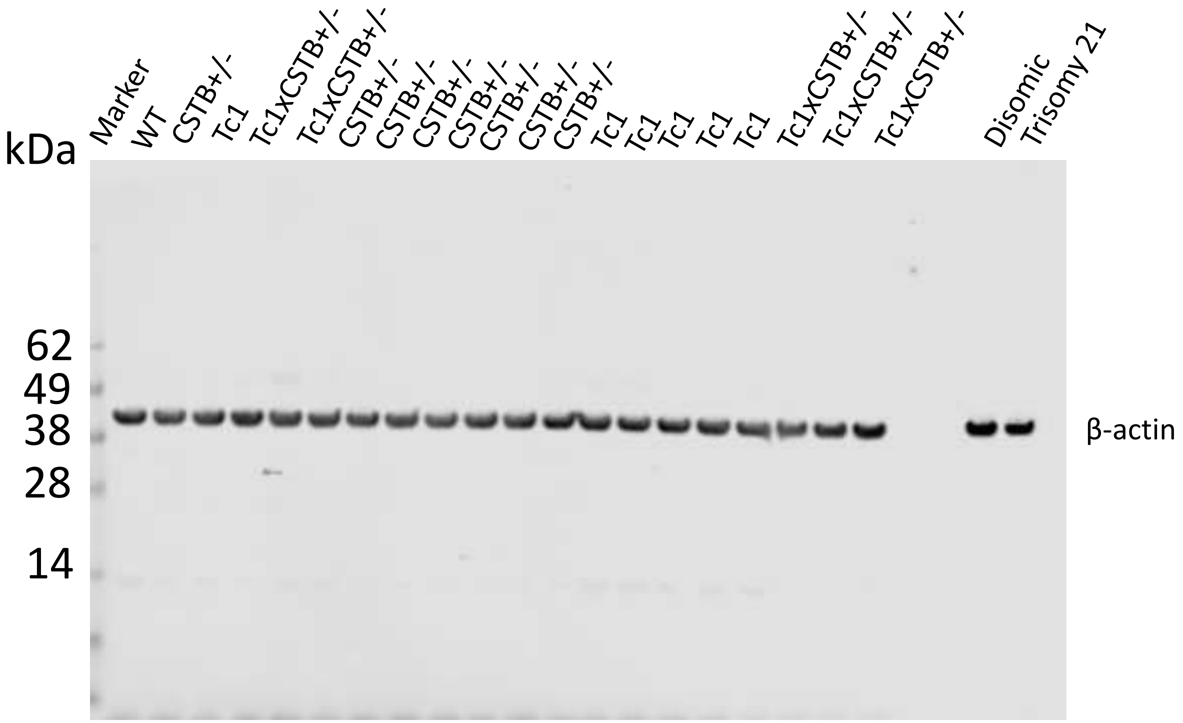

For Figure 3a

PVDF membranes

By Odyssey CLx imaging system (Yixing Wu)

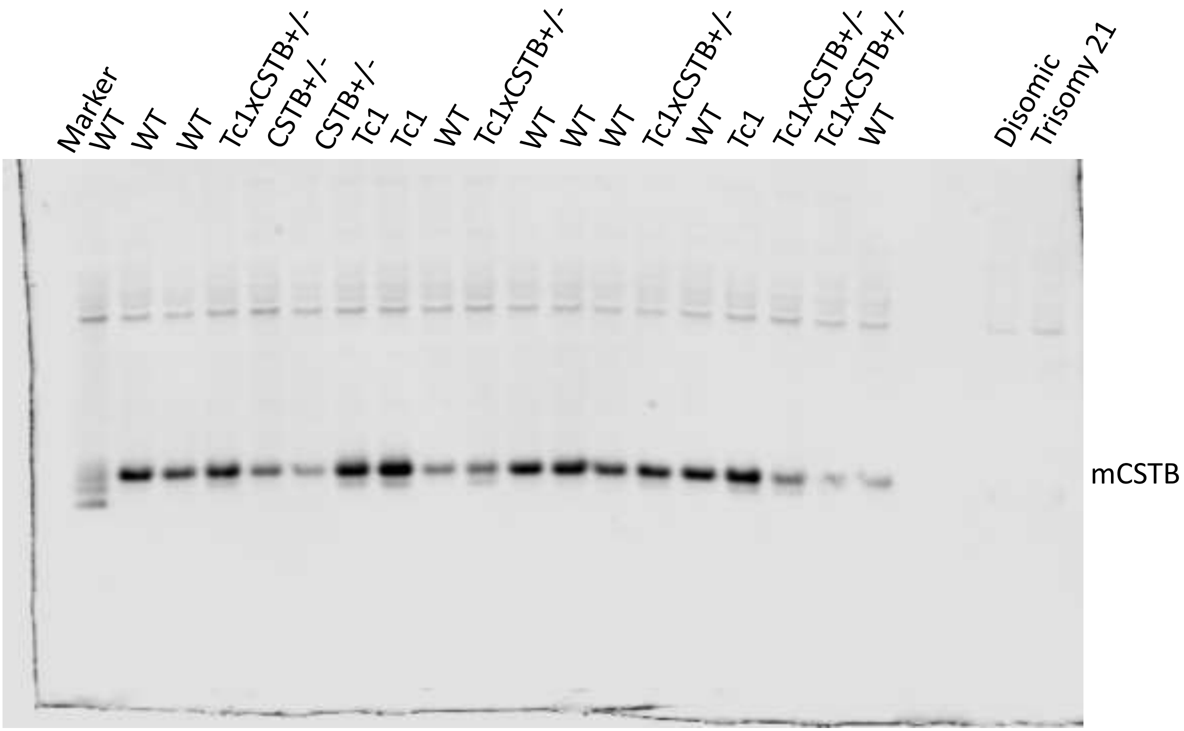

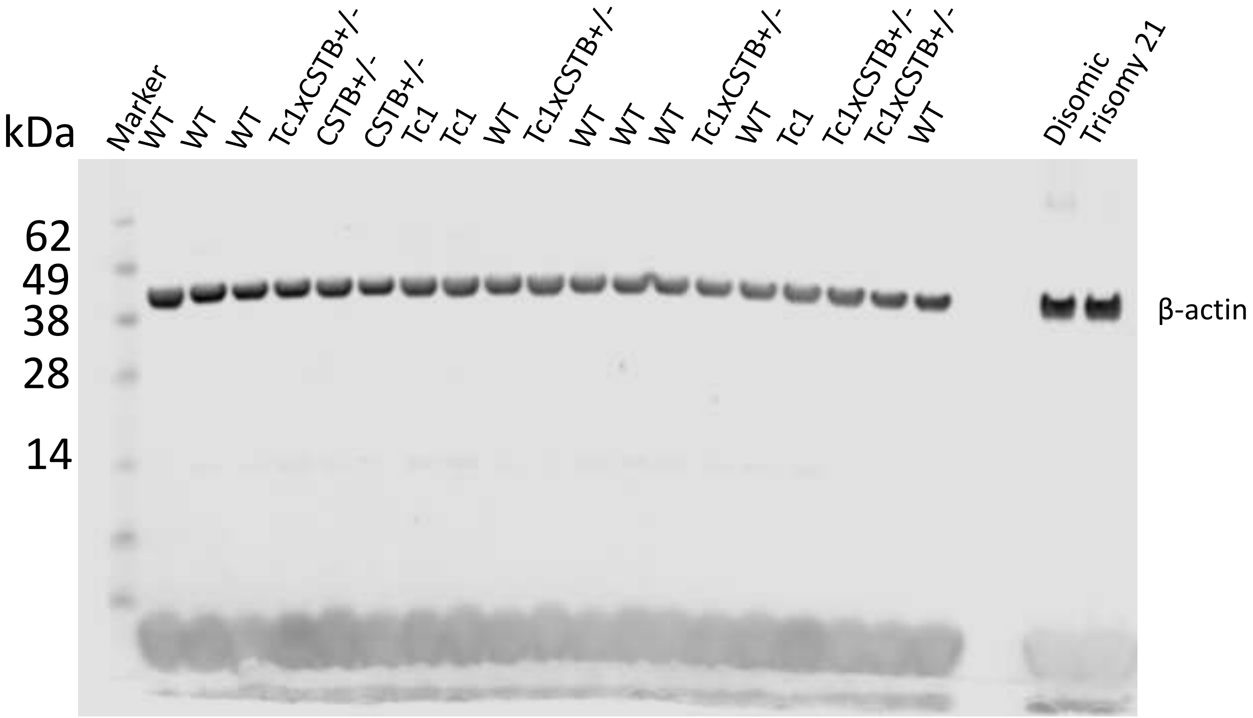

For Figure 3a

PVDF membranes

By Odyssey CLx imaging system (Yixing Wu)

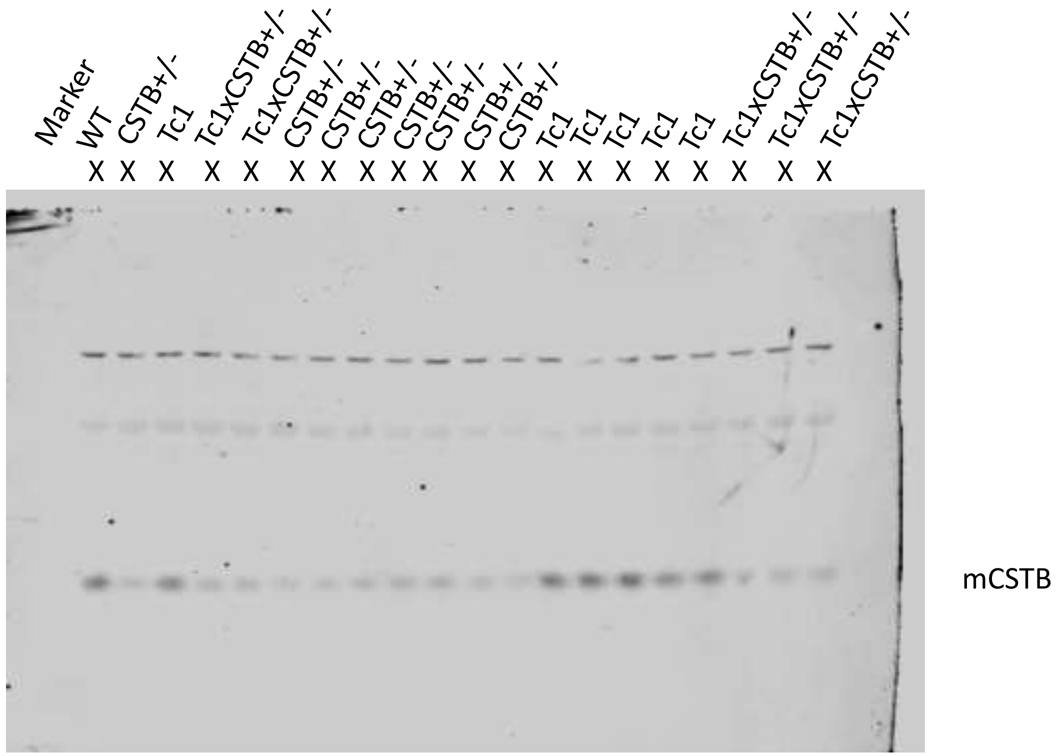

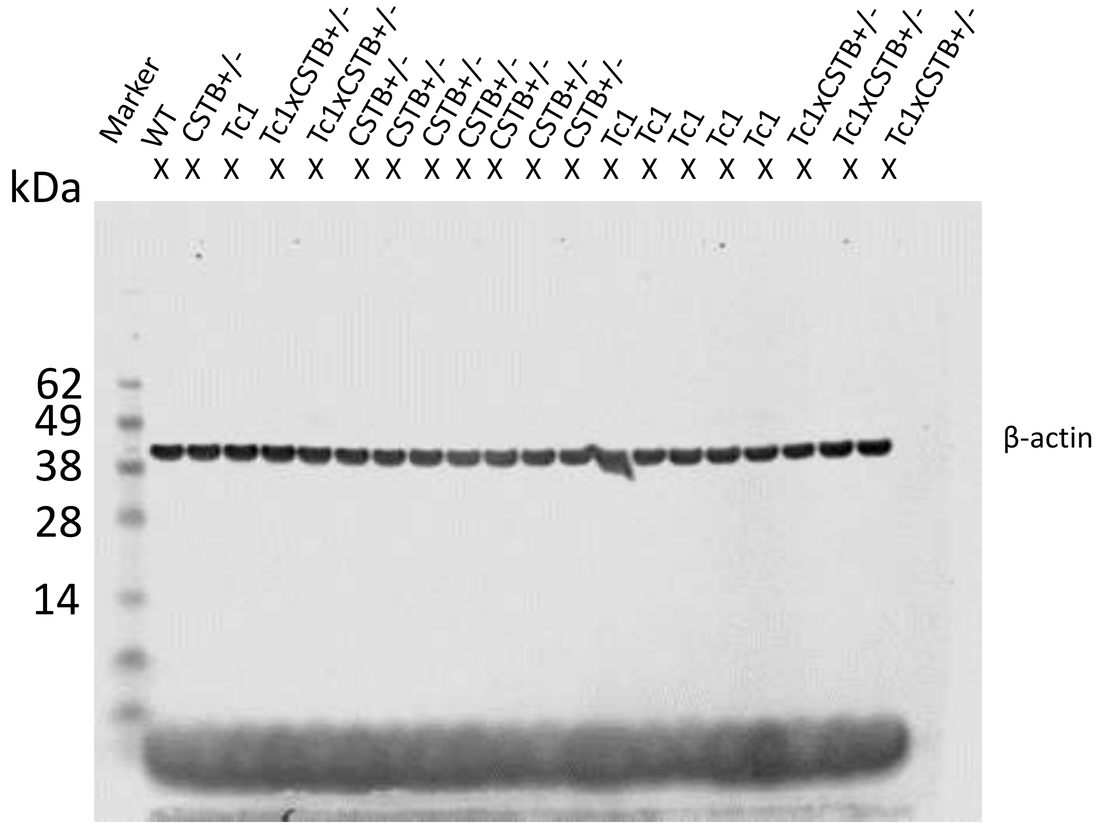

X – indicates lanes from which data was quantitated but that were not included in the representative images in Wu et al 2024.

For Figure 3a

PVDF membranes

By Odyssey CLx imaging system (Yixing Wu)

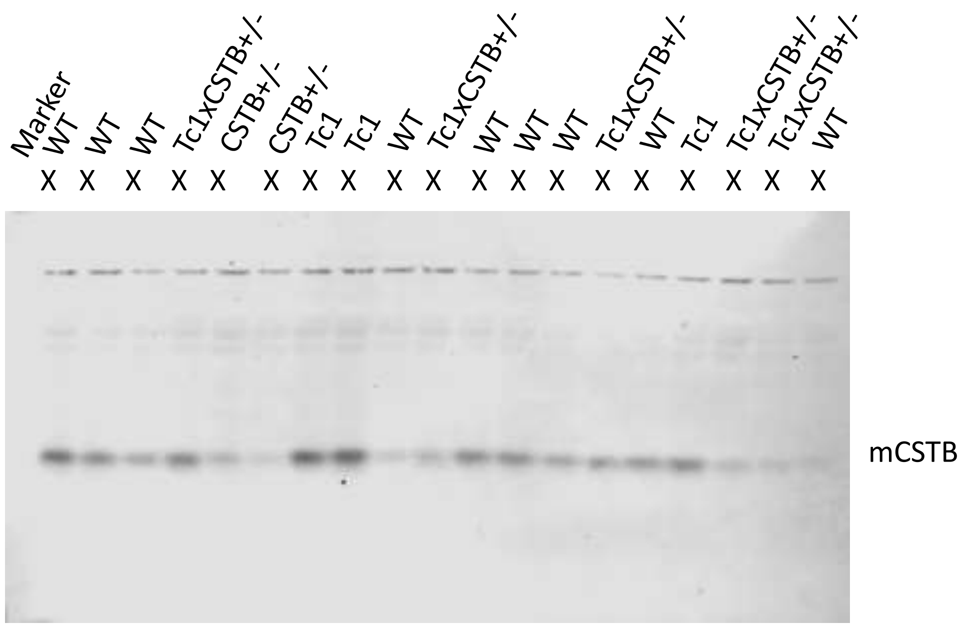

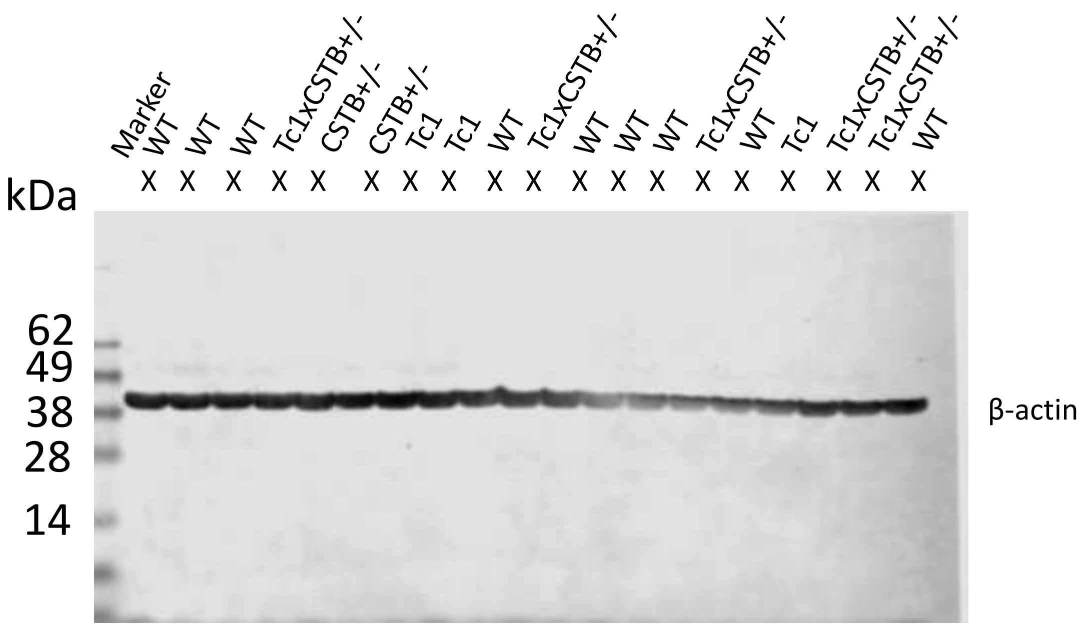

X – indicates lanes from which data was quantitated but that were not included in the representative images in Wu et al 2024.

For Figure 3a

PVDF membranes

By Odyssey CLx imaging system (Yixing Wu)

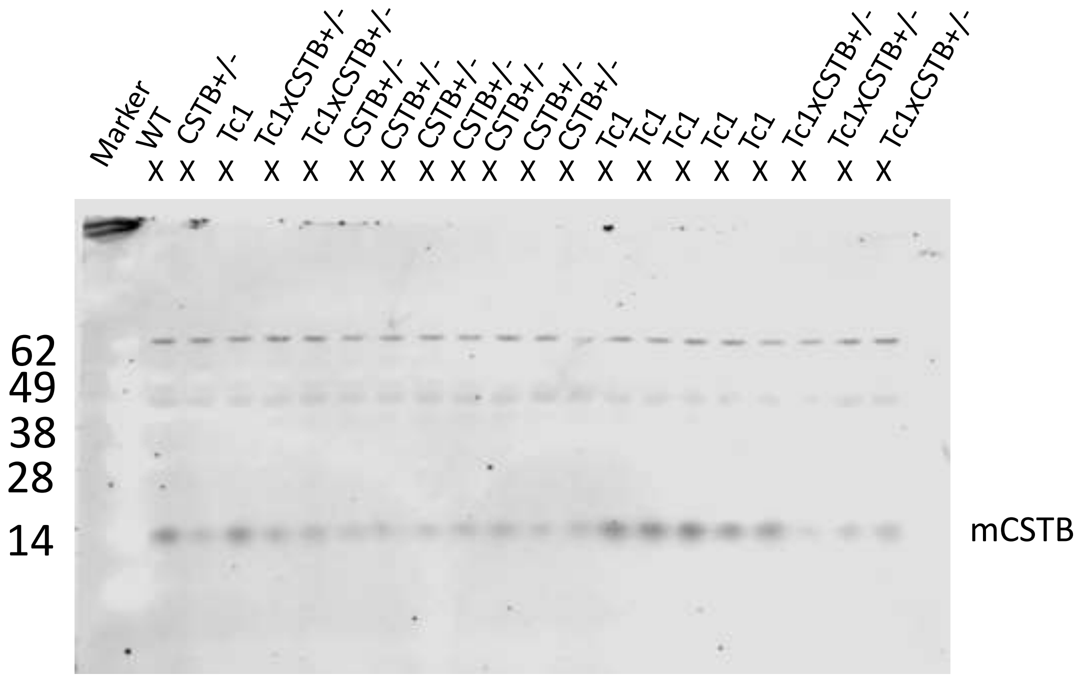

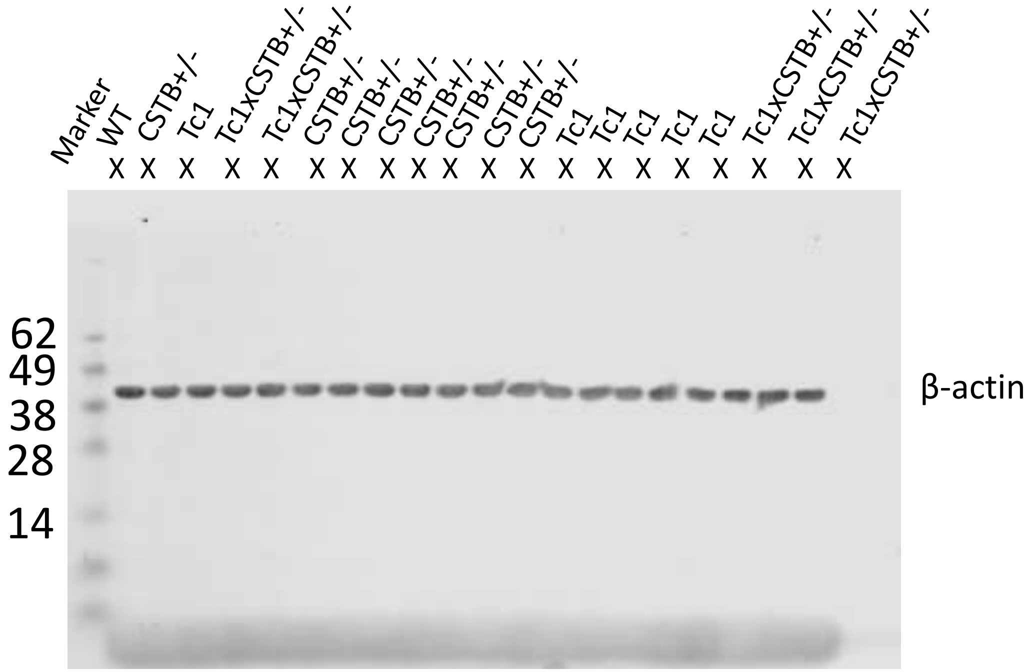

X – indicates lanes from which data was quantitated but that were not included in the representative images in Wu et al 2024.

For Figure 3a

PVDF membranes

By Odyssey CLx imaging system (Yixing Wu)

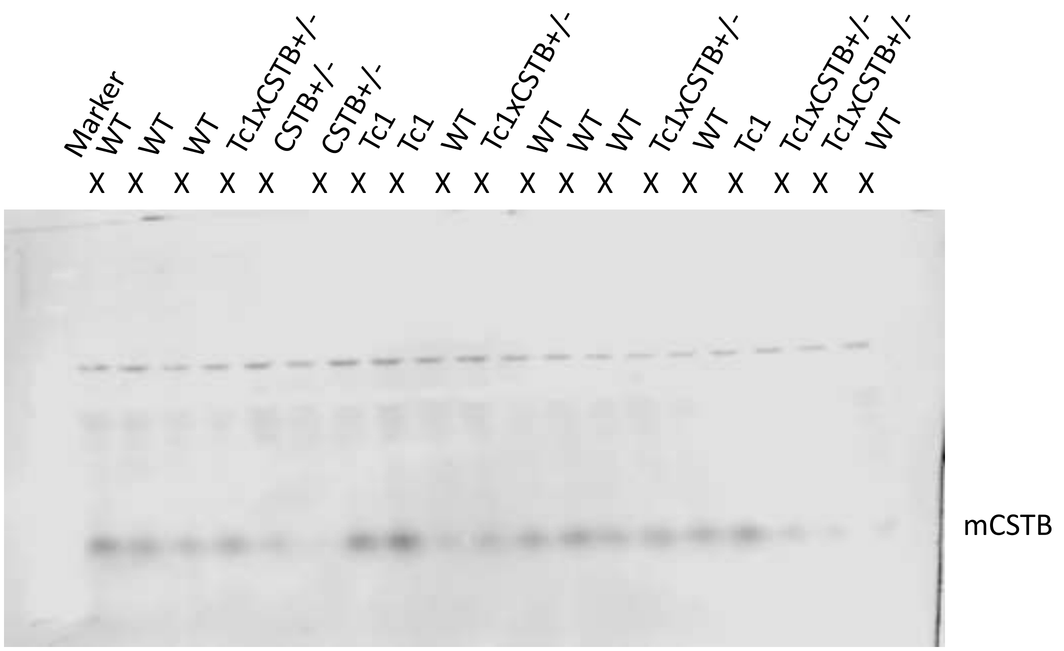

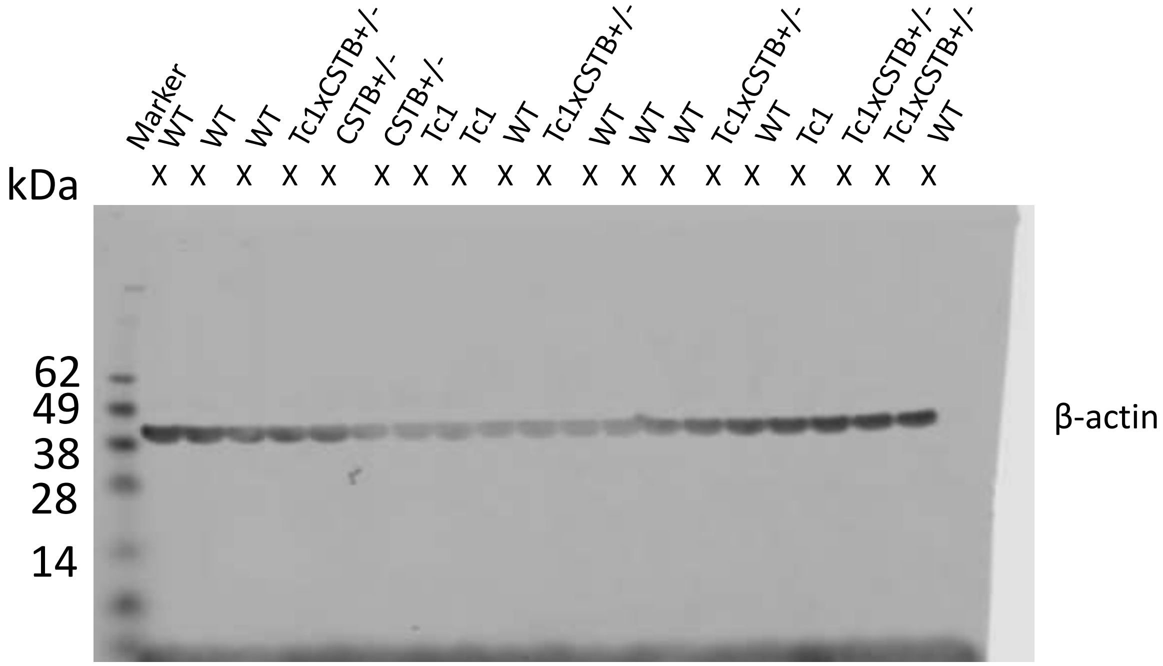

X – indicates lanes from which data was quantitated but that were not included in the representative images in Wu et al 2024.

For Figure 3b

PVDF membranes

By Odyssey CLx imaging system (Yixing Wu)

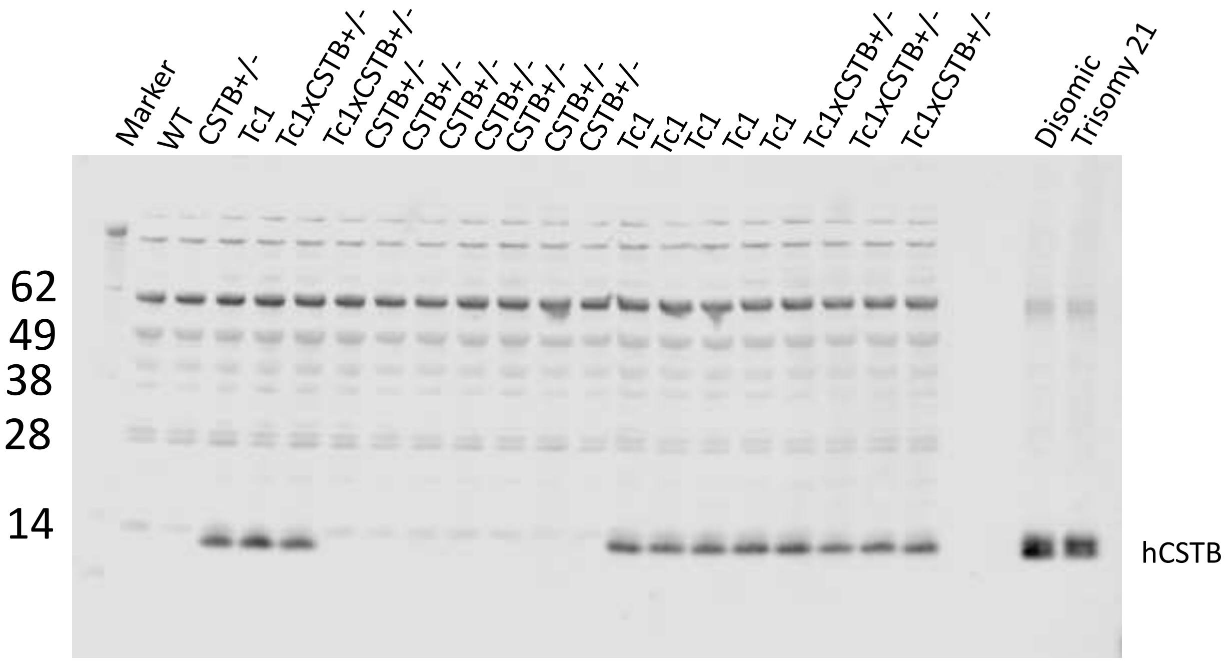

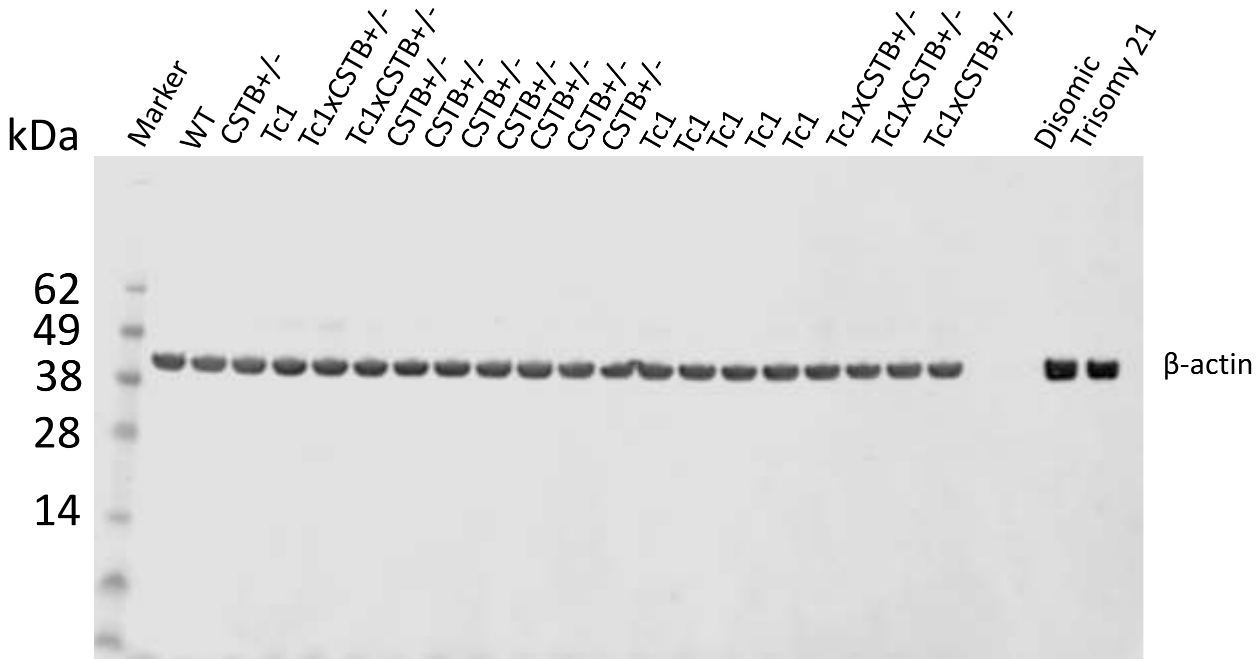

For Figure 3b

PVDF membranes

By Odyssey CLx imaging system (Yixing Wu)

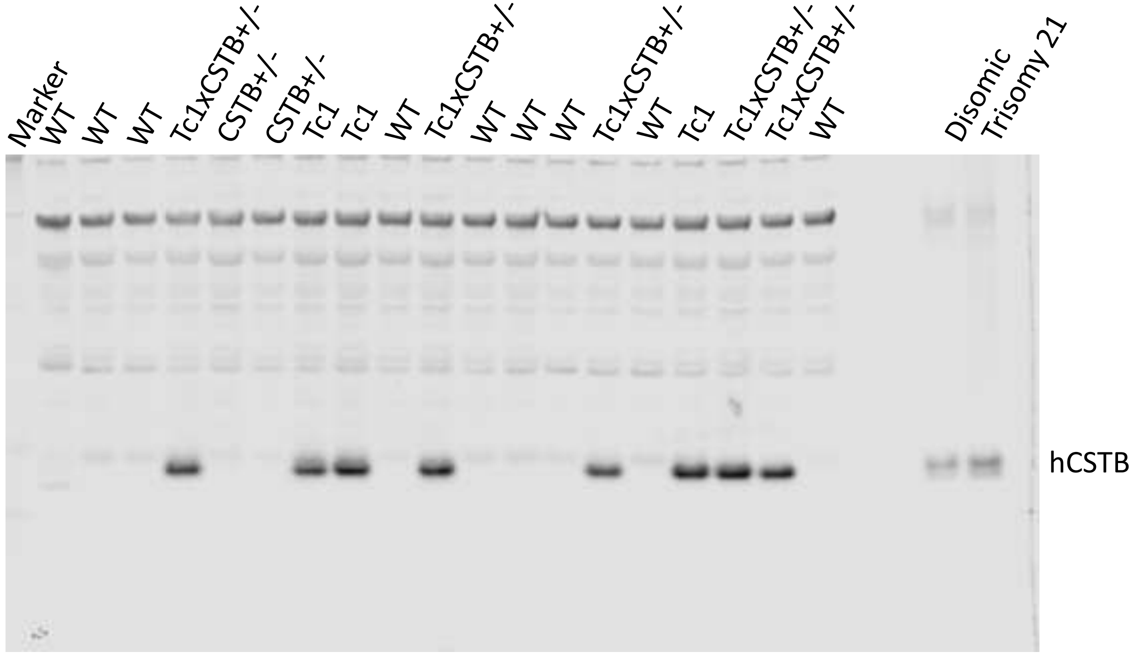

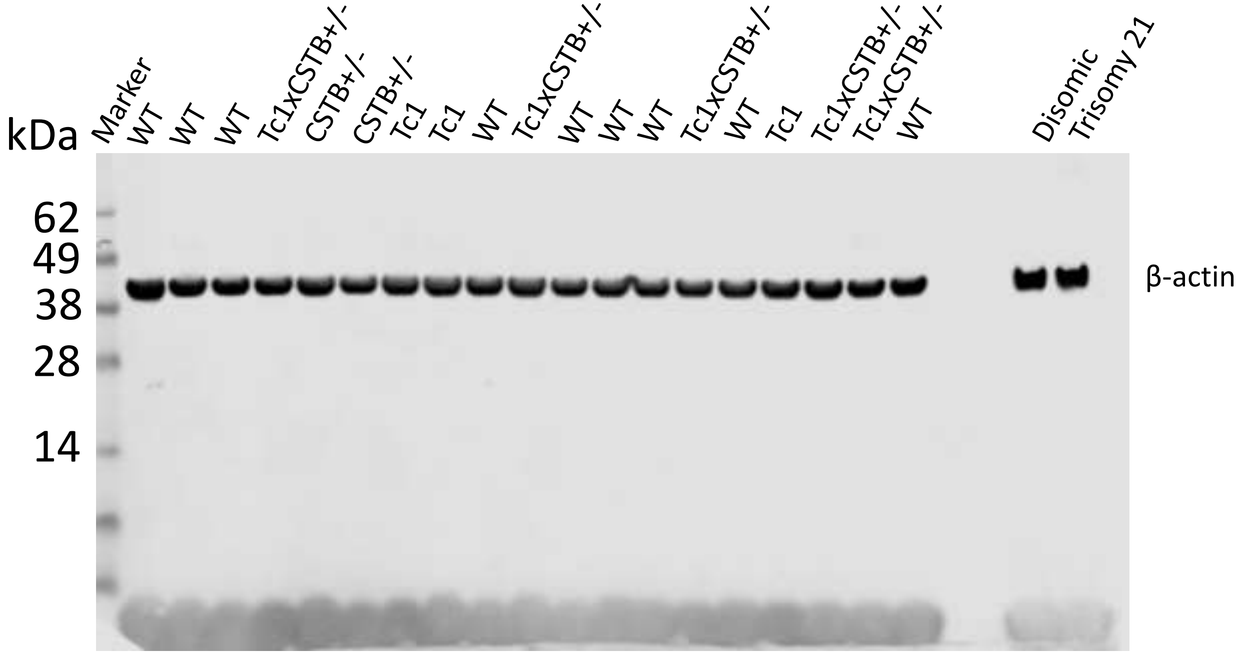

For Figure 3c

PVDF membranes

By Odyssey CLx imaging system (Yixing Wu)

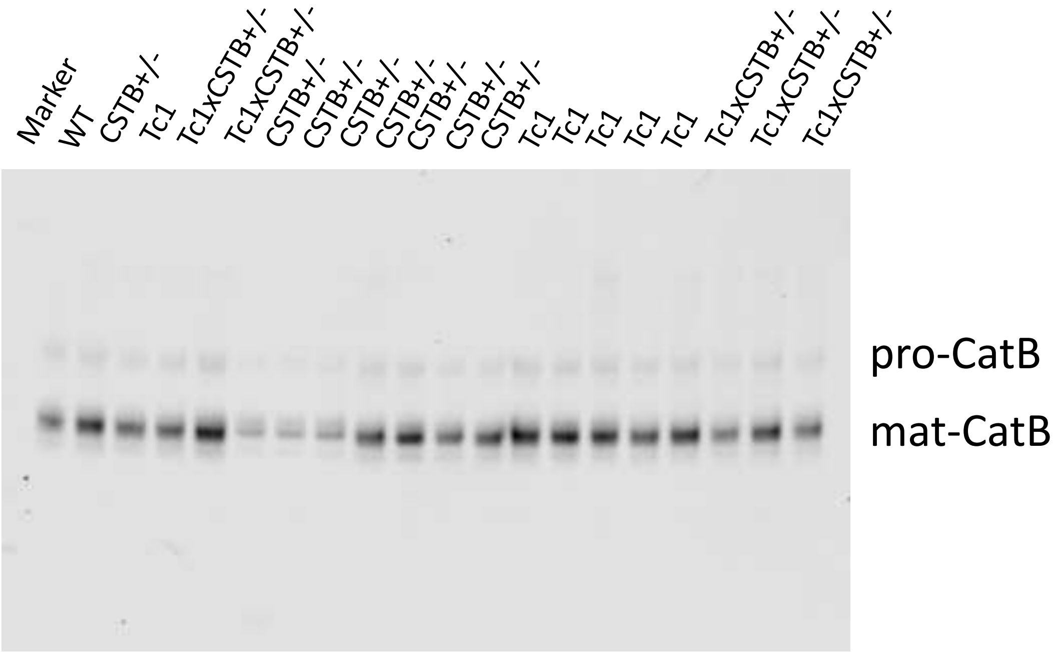

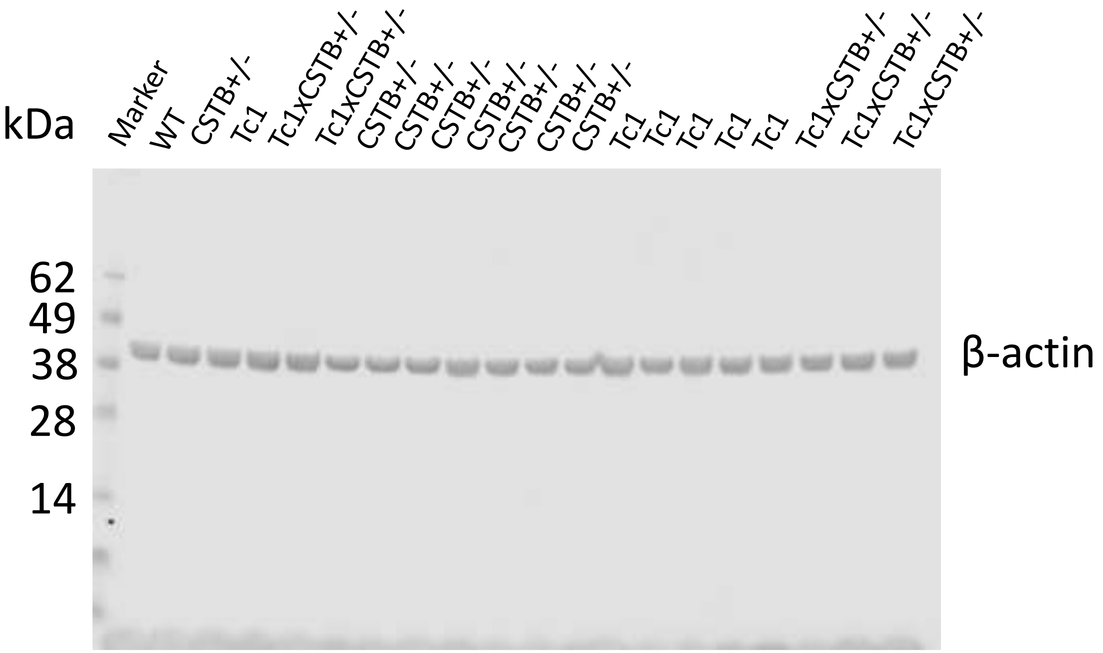

For Figure 3c

PVDF membranes

By Odyssey CLx imaging system (Yixing Wu)

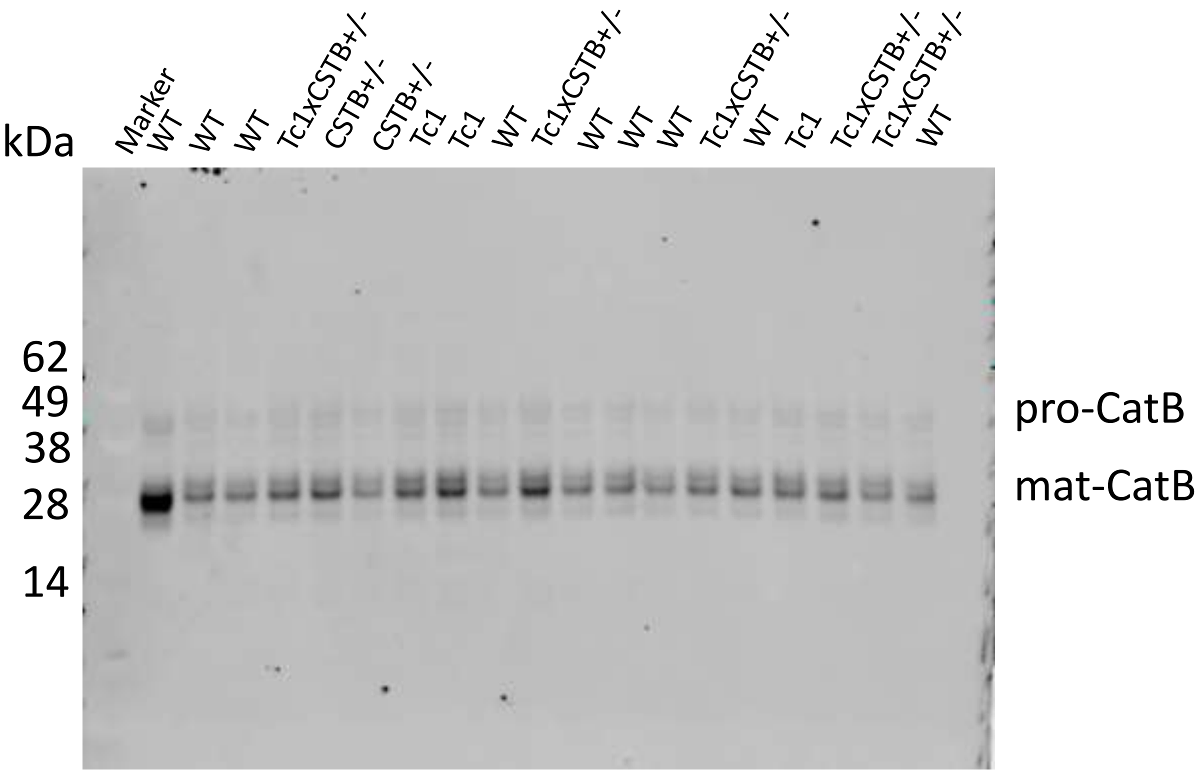

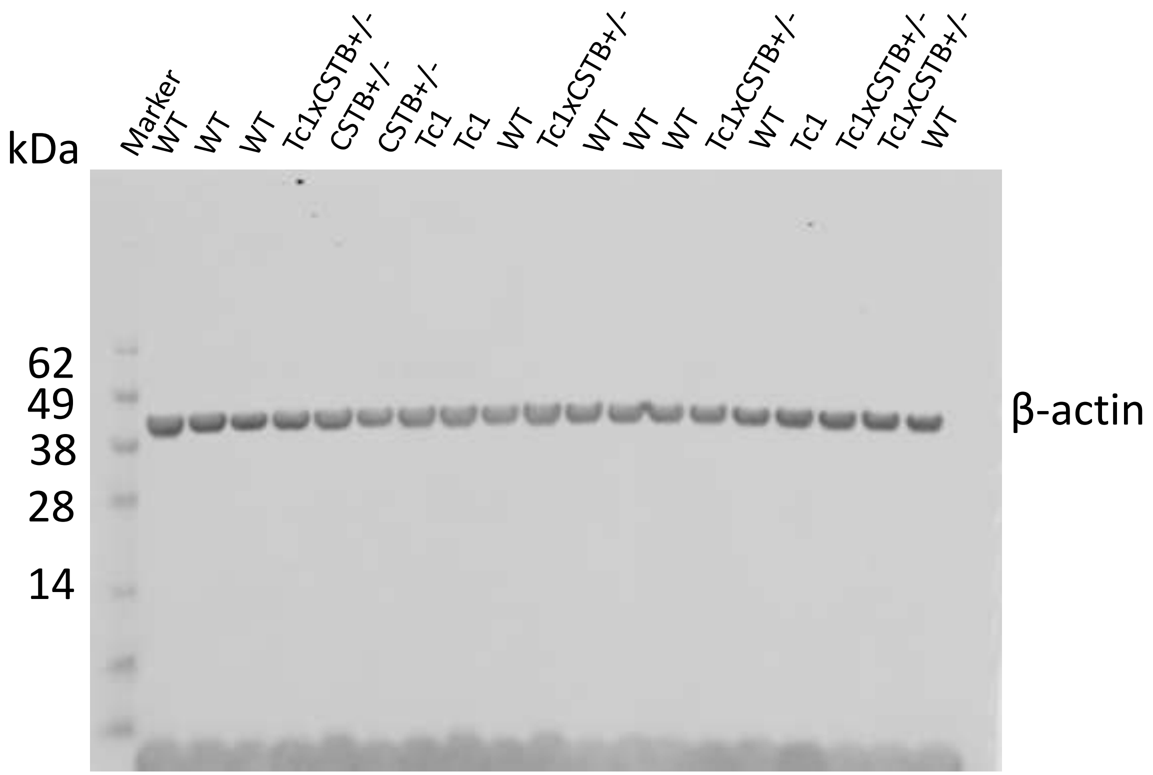

For Figure 3c

PVDF membranes

By Odyssey CLx imaging system (Yixing Wu)

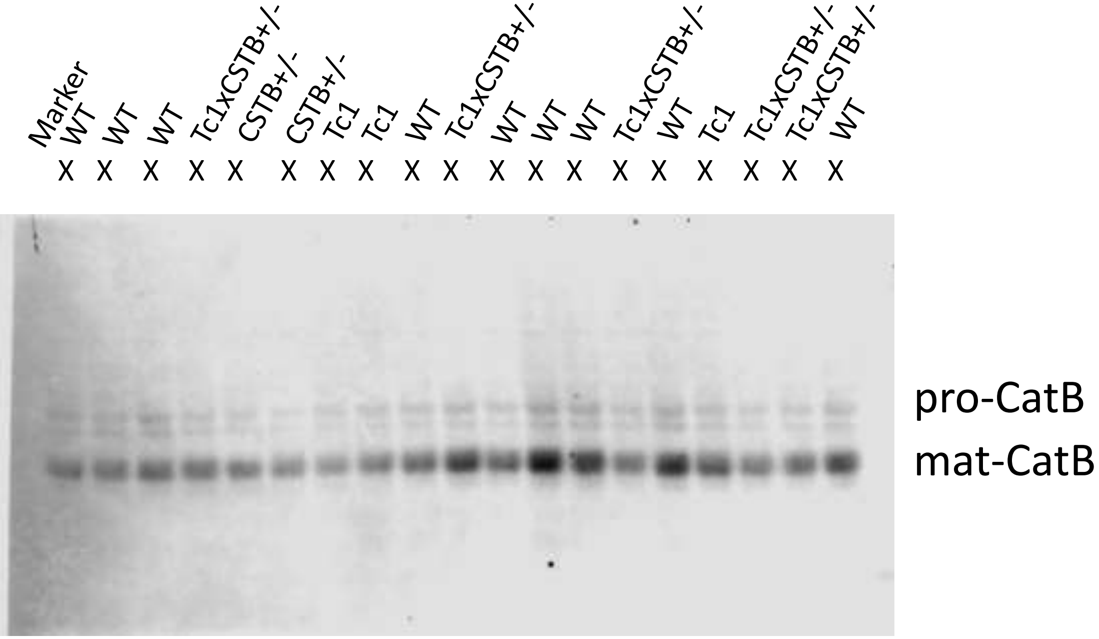

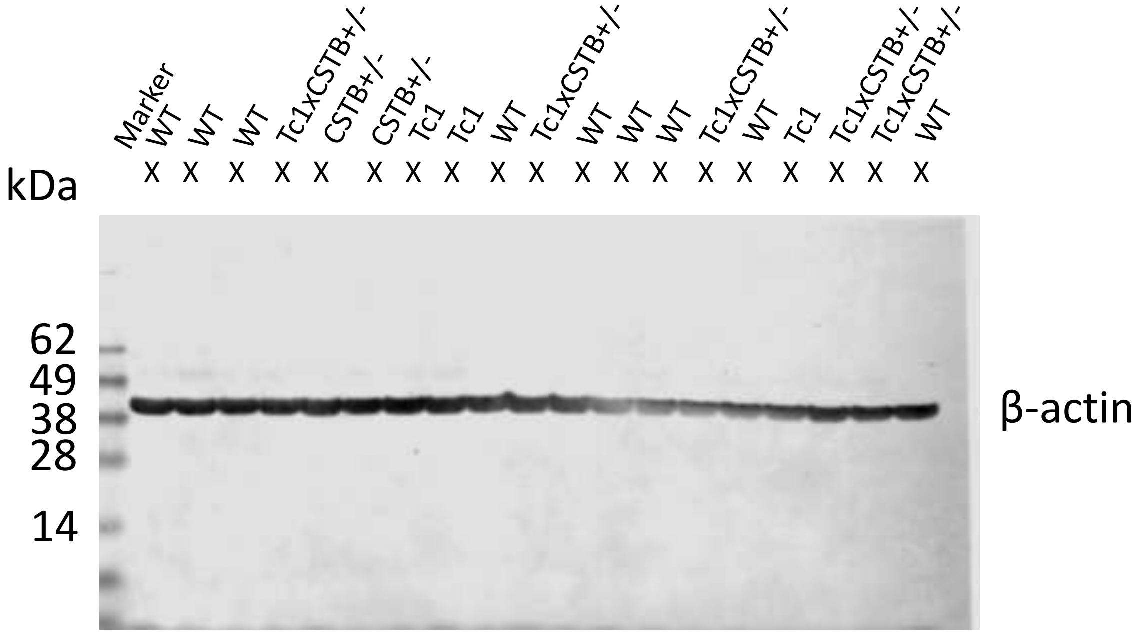

The same blot as the β-actin blot for mCSTB n2

X – indicates lanes from which data was quantitated but that were not included in the representative images in Wu et al 2024.

For Figure 3c

PVDF membranes

By Odyssey CLx imaging system (Yixing Wu)

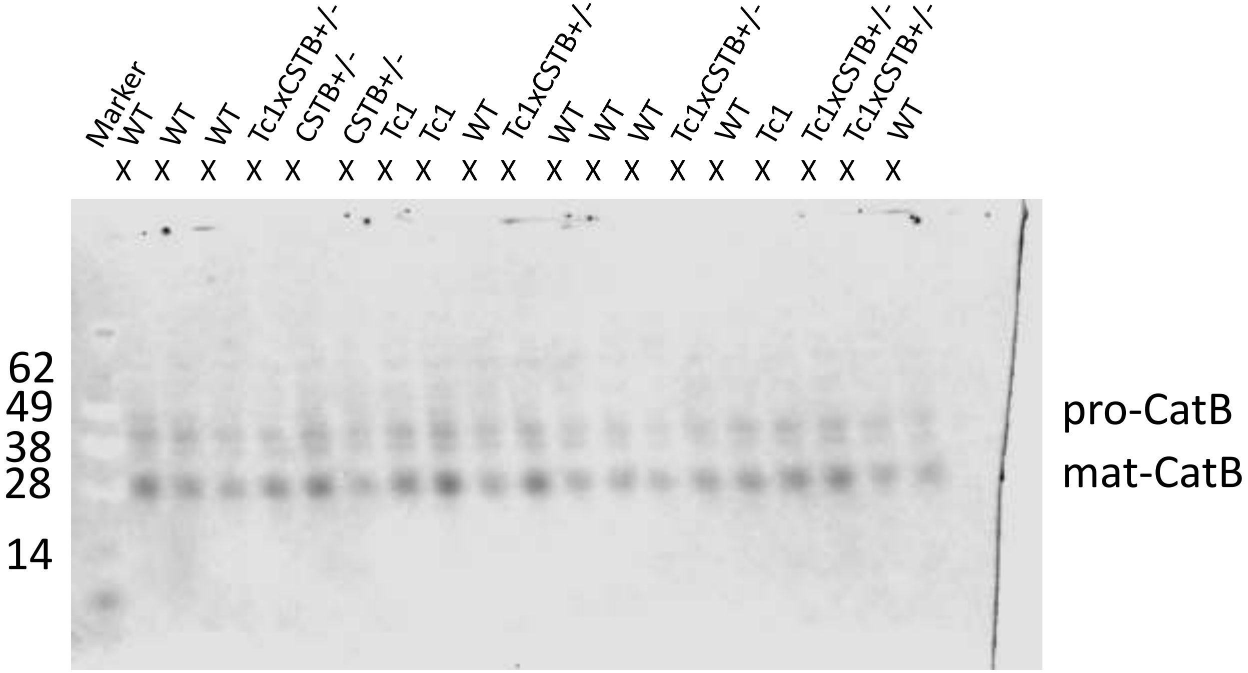

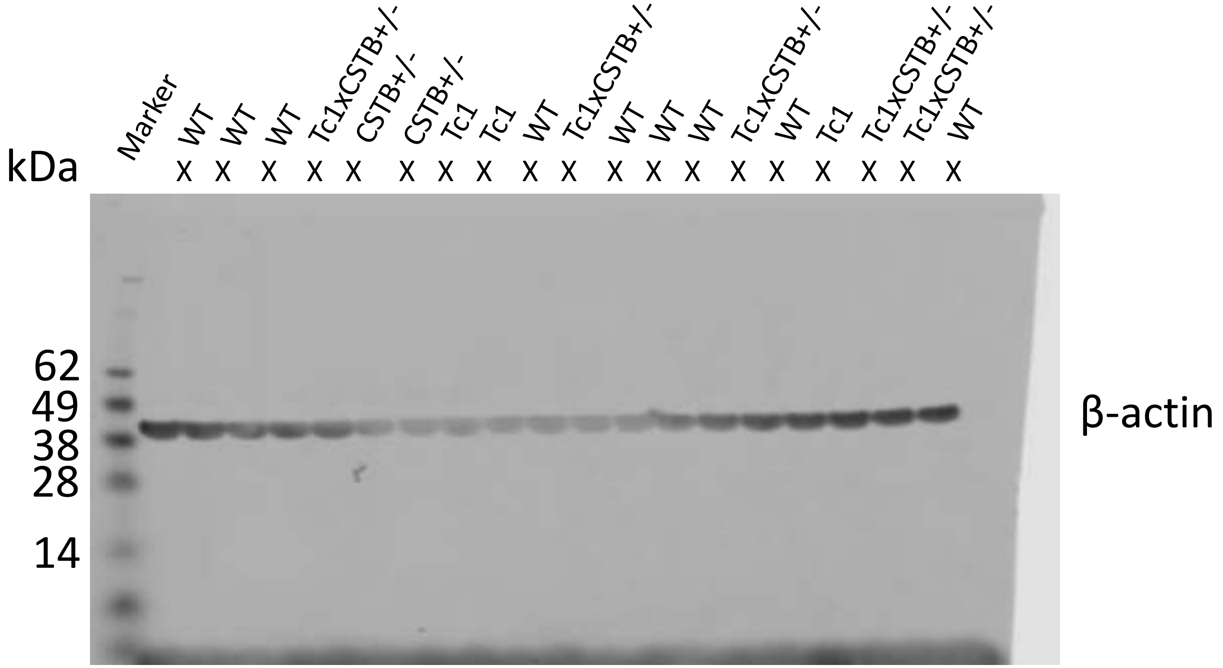

The same blot as the β-actin blot for mCSTB n3

X – indicates lanes from which data was quantitated but that were not included in the representative images in Wu et al 2024.

For Supplementary Figure 1

PVDF membranes

By Odyssey CLx imaging system (Yixing Wu)

1:2000 (1 ul) DharmaFECT

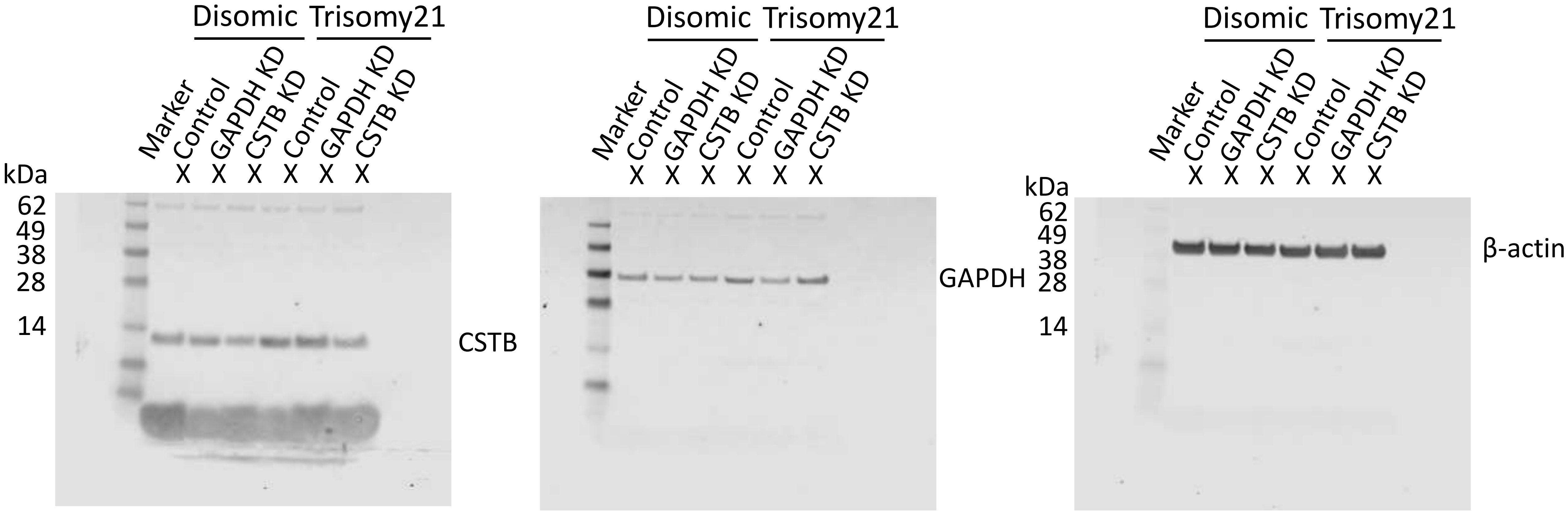

X – indicates lanes from which data was quantitated but that were not included in the representative images in Wu et al 2024.

For Supplementary Figure 1

PVDF membranes

By Odyssey CLx imaging system (Yixing Wu)

1:2000 (1 ul) DharmaFECT

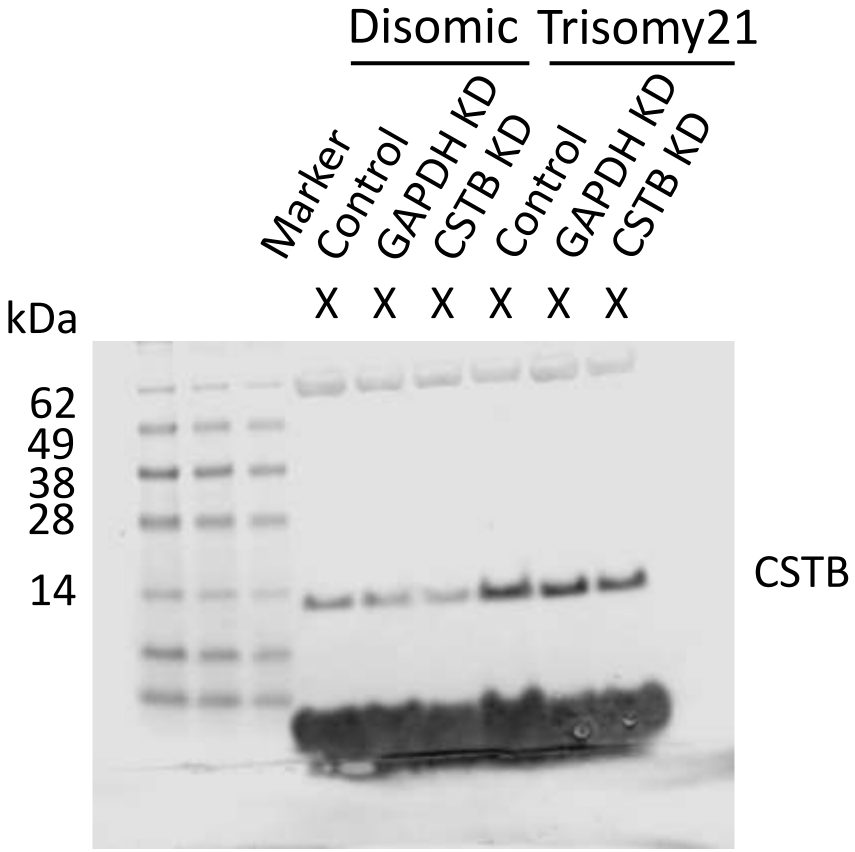

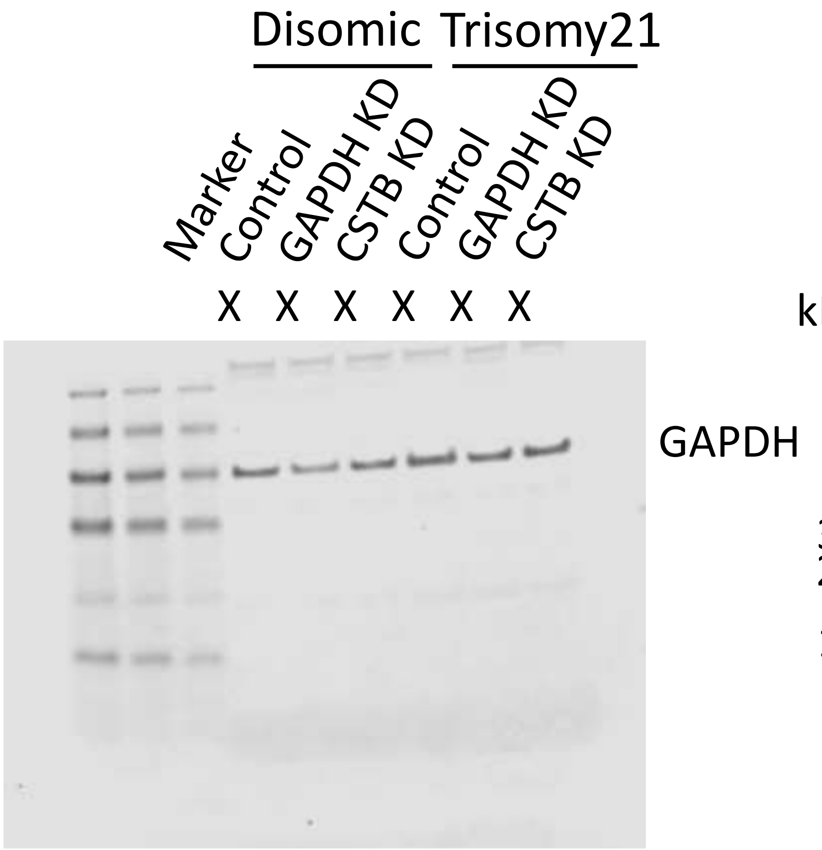

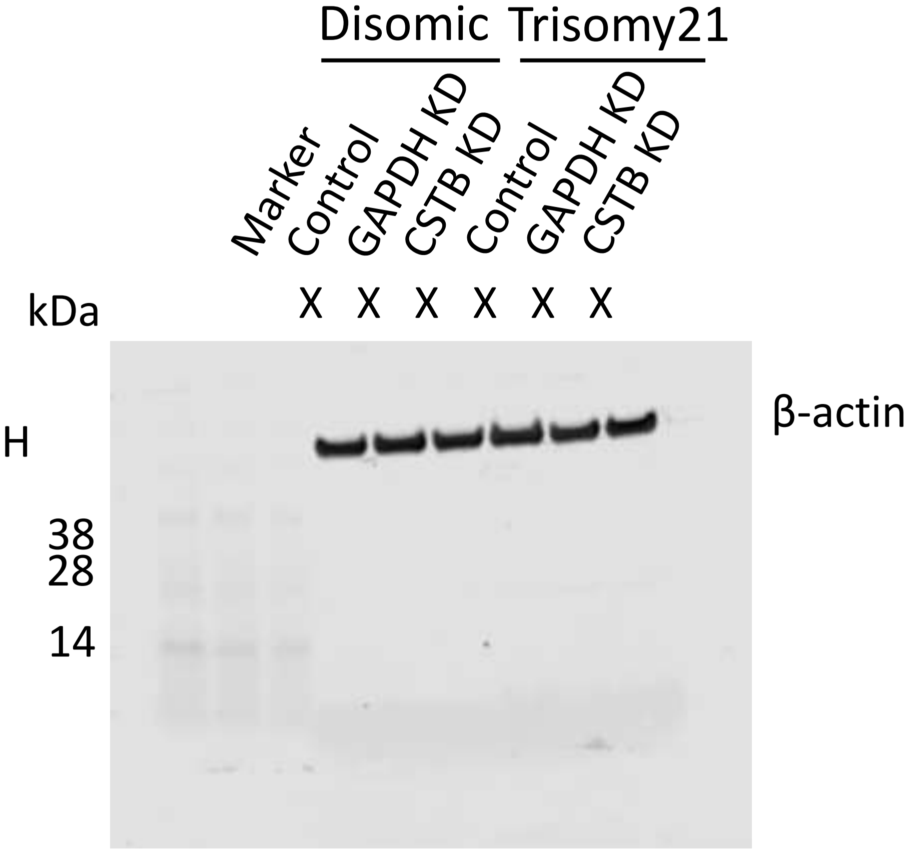

X – indicates lanes from which data was quantitated but that were not included in the representative images in Wu et al 2024.

For Supplementary Figure 1

PVDF membranes

By Odyssey CLx imaging system (Yixing Wu)

1:2000 (1 ul) DharmaFECT

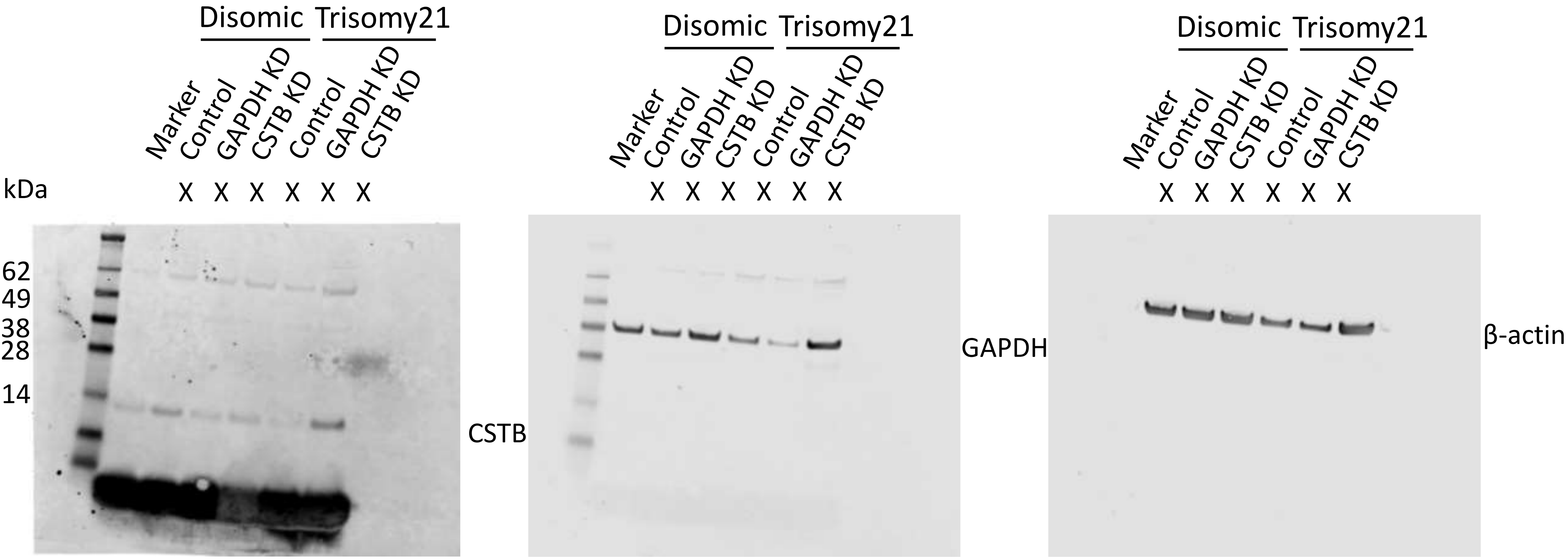

X – indicates lanes from which data was quantitated but that were not included in the representative images in Wu et al 2024.

For Supplementary Figure 1

PVDF membranes

By Odyssey CLx imaging system (Yixing Wu)

1:800 (2.5 ul) DharmaFECT

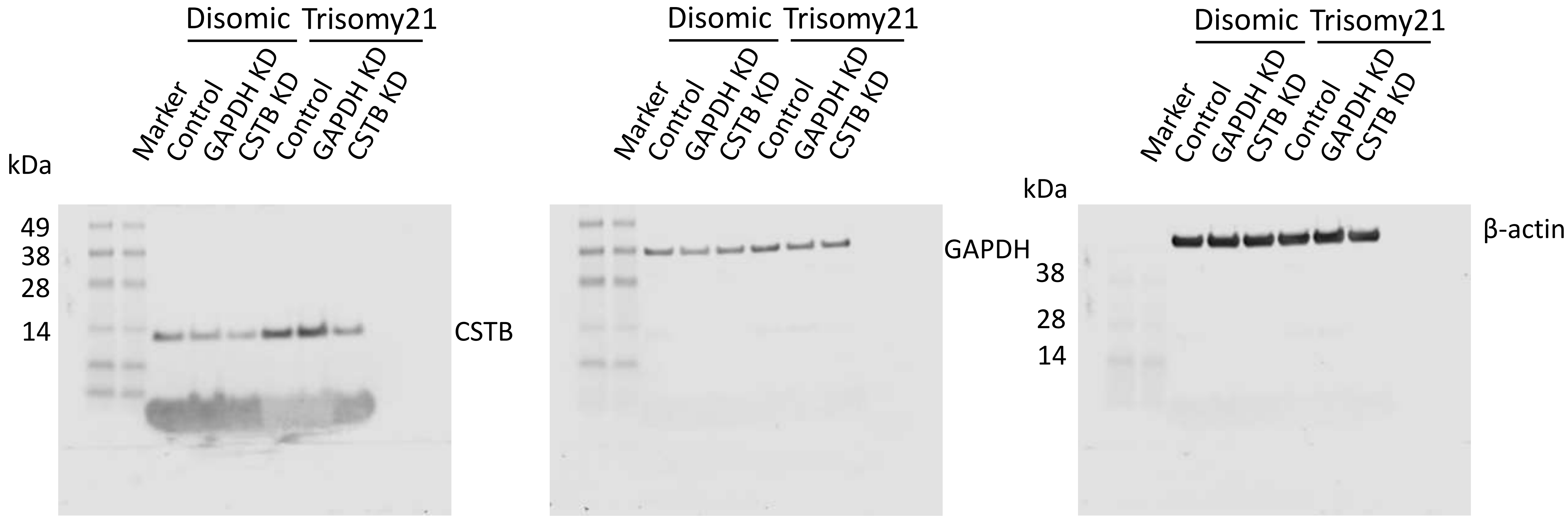

X – indicates lanes from which data was quantitated but that were not included in the representative images in Wu et al 2024.

For Supplementary Figure 1

PVDF membranes

By Odyssey CLx imaging system (Yixing Wu)

1:800 (2.5 ul) DharmaFECT

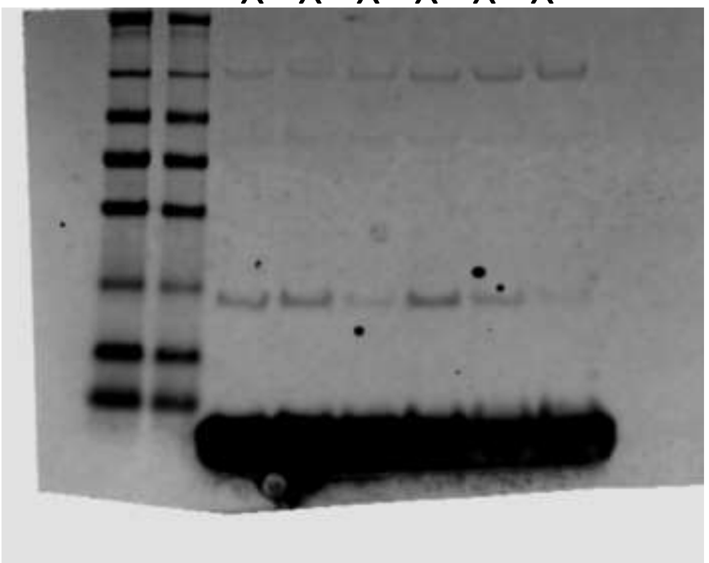

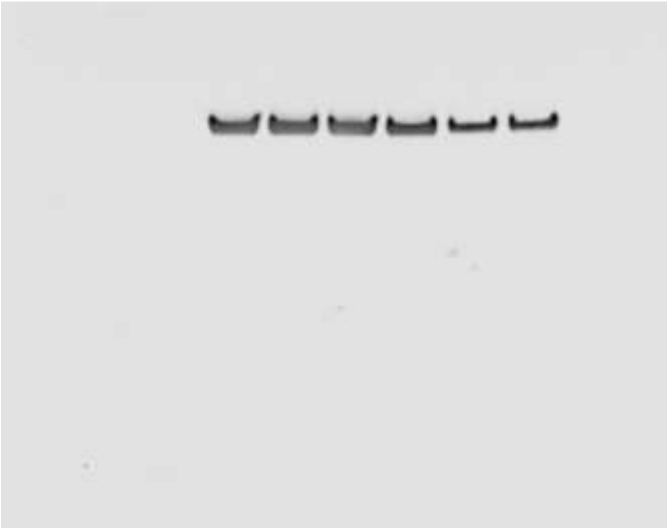

1

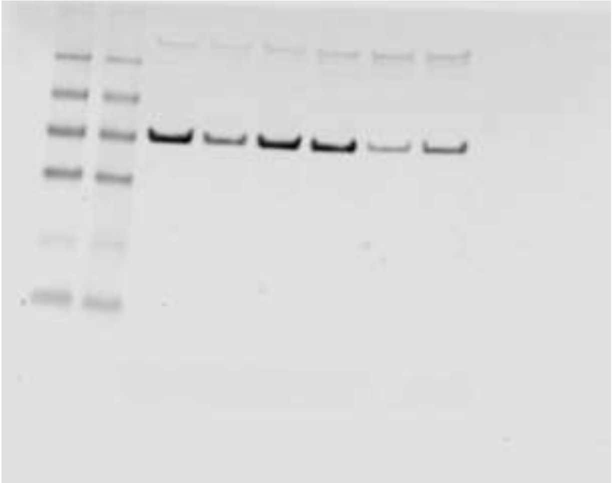

2

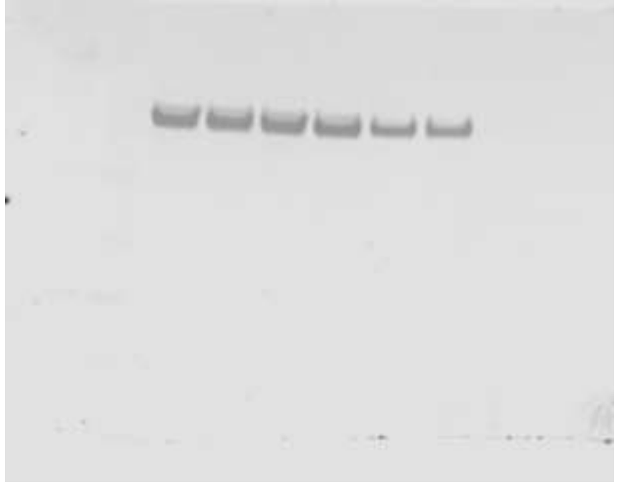

1 and 2 are both from the same membrane

X – indicates lanes from which data was quantitated but that were not included in the representative images in Wu et al 2024.

For Supplementary Figure 1

PVDF membranes

By Odyssey CLx imaging system (Yixing Wu)

1:800 (2.5 ul) DharmaFECT

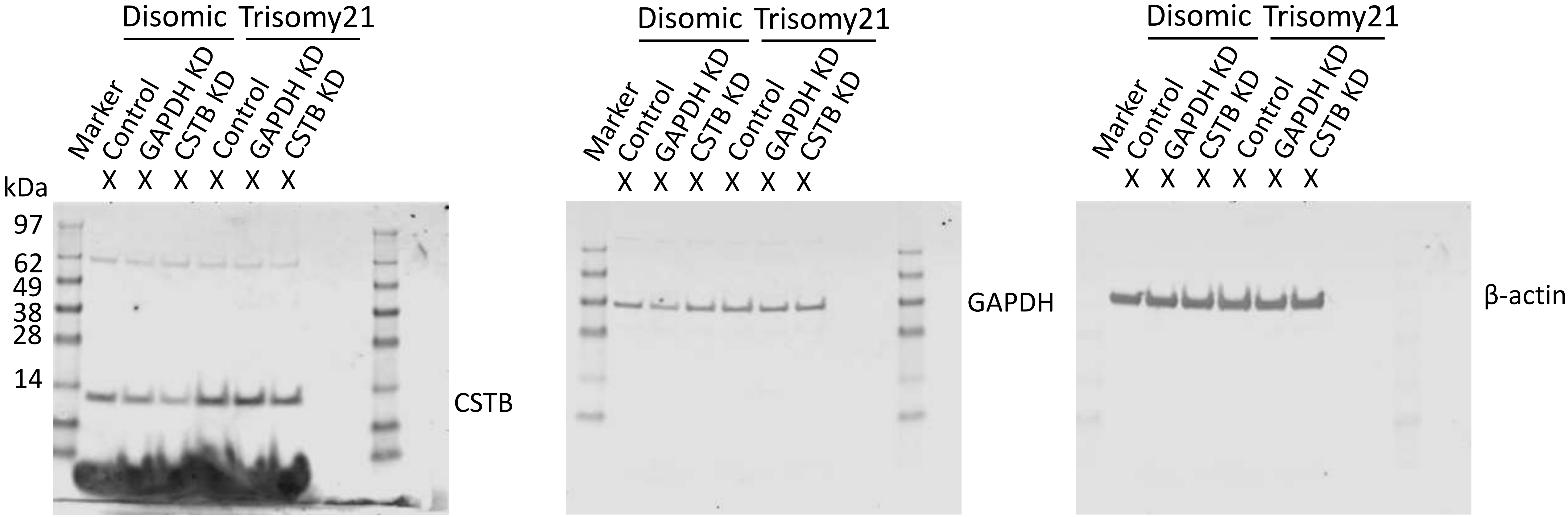

X – indicates lanes from which data was quantitated but that were not included in the representative images in Wu et al 2024.

For Supplementary Figure 1

PVDF membranes

By Odyssey CLx imaging system (Yixing Wu)

1:2000 (1 ul) or 1:800 (2.5 ul) DharmaFECT

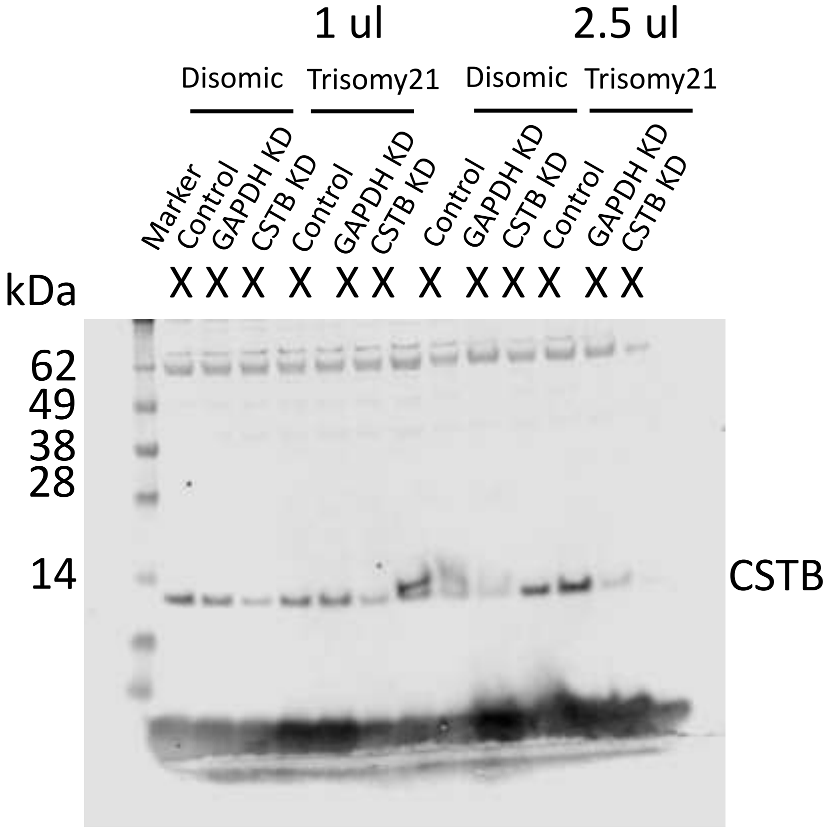

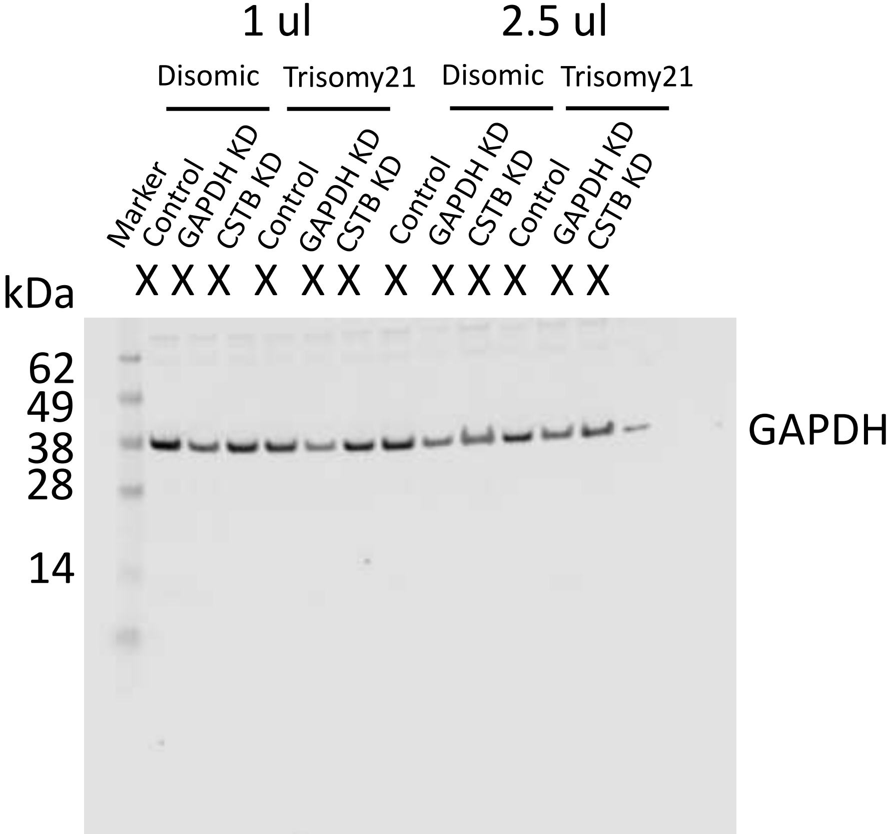

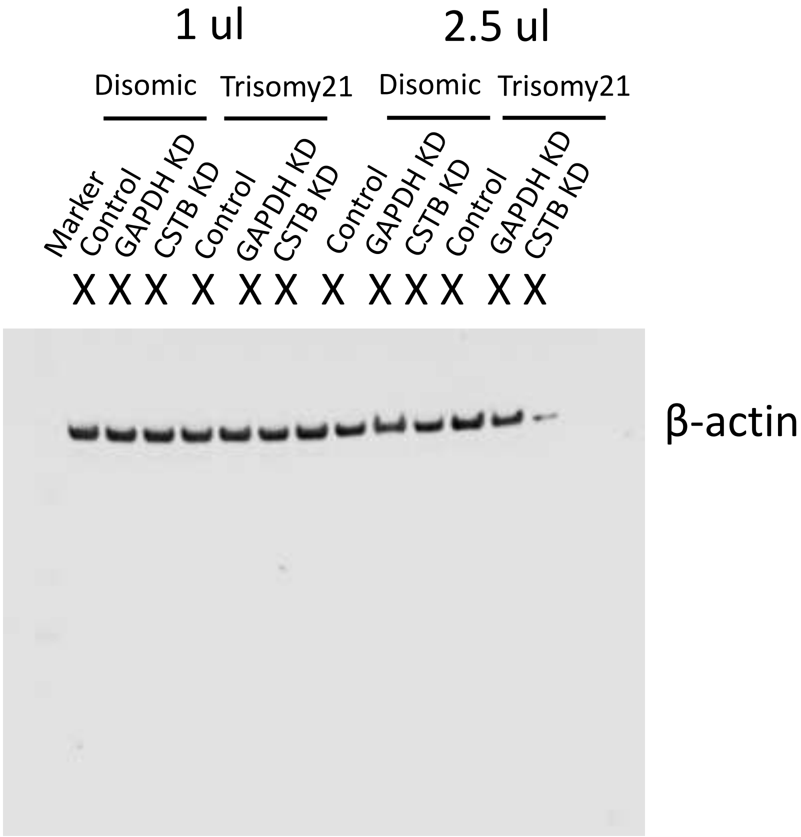

X – indicates lanes from which data was quantitated but that were not included in the representative images in Wu et al 2024.
