# Supplementary figures and images for: Reduction of Cystatin B results in increased cathepsin B activity in disomic but not Trisomy 21 human cellular and mouse models
Source: PLoS One. 2025 Jan 22;20(1):e0316822. doi: 10.1371/journal.pone.0316822 (PMC11753708; doi:10.1371/journal.pone.0316822)

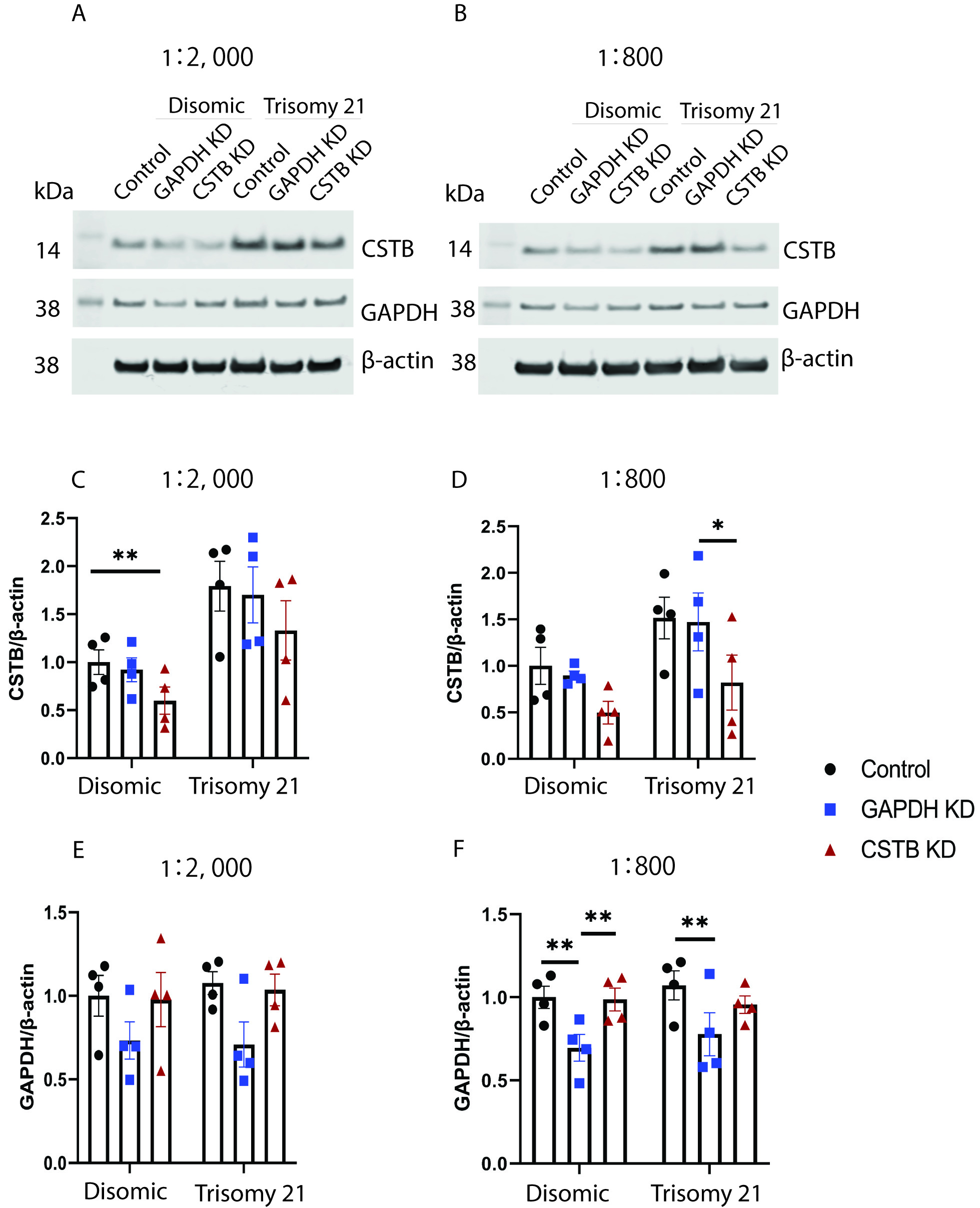

Supplement: S2 Fig — (A-B) Western blot of CSTB and GAPDH normalised to β-actin in disomic and trisomy 21 human fibroblasts in (A) 1:2,000 dilution of DharmaFECT reagent or (B) 1:800 dilution DharmaFECT reagent. (C) 1:2,000 dilution of DharmaFECT-mediated CSTB knockdown reduced CSTB abundance compared with the control group (p = 0.0058) in the disomic, but not in the trisomy 21 fibroblasts. (D) 1:800 dilution of DharmaFECT-mediated CSTB knockdown reduced CSTB abundance in the CSTB KD group compared with the GAPDH KD group (p = 0.0139), but not compared to the control group in the trisomic 21 human fibroblasts or the disomic group. (E) 1:2000 dilution of DharmaFECT-mediated GAPDH knockdown does not affect GAPDH abundance in either disomic or trisomy 21 fibroblasts. (F) 1:800 dilution of DharmaFECT-mediated GAPDH knockdown reduced GAPDH abundance in disomic fibroblasts compared to control (p = 0.002) and CSTB (p = 0.0028) groups. In the trisomy 21 fibroblasts, GAPDH knockdown reduced GAPDH abundance compared with the control (p = 0.0025), but not the CSTB knockdown group. Data are shown as ±SEM of group means for 4 disomic and 4 trisomy 21 lines (1 technical replicate for western blots). Data were analysed by two-way ANOVA followed by Tukey’s post-hoc tests, *p<0.05, ***p<0.001, ****p<0.0001. (TIF) [file pone.0316822.s002.tif]
